# The first whole transcriptomic exploration of pre-oviposited early chicken embryos using single and bulked embryonic RNA-sequencing

**DOI:** 10.1093/gigascience/giy030

**Published:** 2018-03-24

**Authors:** Young Sun Hwang, Minseok Seo, Hee Jung Choi, Sang Kyung Kim, Heebal Kim, Jae Yong Han

**Affiliations:** 1 Department of Agricultural Biotechnology and Research Institute of Agriculture and Life Sciences, Seoul National University, Seoul 08826, Korea; 2 CHO&KIM genomics, SNU Research Park, Seoul National University Mt.4-2, Seoul 08826, Korea; 3 Channing Division of Network Medicine, Harvard Medical School and Brigham and Women's Hospital, Boston, Massachusetts, USA; 4 Institute for Biomedical Sciences, Shinshu University, Minamiminowa, Nagano 399-4598, Japan

**Keywords:** RNA-seq, single embryonic sequencing, single cell sequencing, early embryo, chicken

## Abstract

**Background:**

The chicken is a valuable model organism, especially in evolutionary and embryology research because its embryonic development occurs in the egg. However, despite its scientific importance, no transcriptome data have been generated for deciphering the early developmental stages of the chicken because of practical and technical constraints in accessing pre-oviposited embryos.

**Findings:**

Here, we determine the entire transcriptome of pre-oviposited avian embryos, including oocyte, zygote, and intrauterine embryos from Eyal-giladi and Kochav stage I (EGK.I) to EGK.X collected using a noninvasive approach for the first time. We also compare RNA-sequencing data obtained using a bulked embryo sequencing and single embryo/cell sequencing technique. The raw sequencing data were preprocessed with two genome builds, Galgal4 and Galgal5, and the expression of 17,108 and 26,102 genes was quantified in the respective builds. There were some differences between the two techniques, as well as between the two genome builds, and these were affected by the emergence of long intergenic noncoding RNA annotations.

**Conclusion:**

The first transcriptome datasets of pre-oviposited early chicken embryos based on bulked and single embryo sequencing techniques will serve as a valuable resource for investigating early avian embryogenesis, for comparative studies among vertebrates, and for novel gene annotation in the chicken genome.

## Background

Avian species are valuable animal models in many research areas, especially in embryology, because the avian embryo develops in an egg before hatching. This is an excellent *in vitro*-like *in vivo* system that has allowed extensive research of the developmental events during embryogenesis. Previous studies have examined primitive streak formation and gastrulation after oviposition in avian species [1–4]. Nevertheless, despite the importance of the initial events in avian embryogenesis before oviposition, only a few morphological studies have examined pre-oviposited embryos because of practical difficulties accessing the embryos [5–7]. The temporal regulation of gene expression during the pre-oviposited stages is important for understanding early embryonic development.

**Figure 1:**
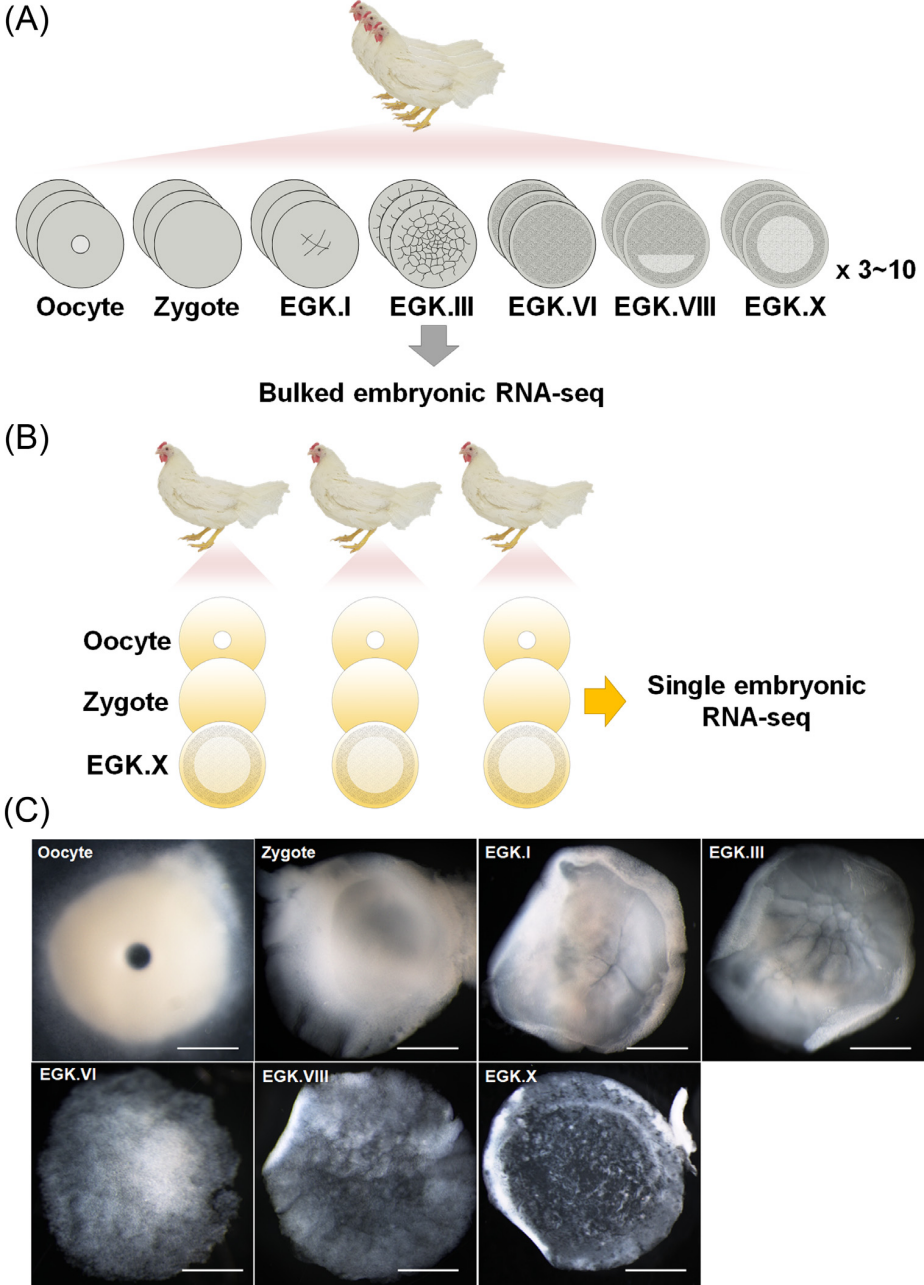
The bulked and single embryonic RNA-sequencing (RNA-seq) in early chicken development. A) The diagram of bulked embryonic RNA-seq. A total of 137 pre-oviposited embryos were collected. Each replicate contains from 3 to 10 embryos pooled. The bulked embryo RNA-seq was performed in triplicate. B) The diagram of single embryonic RNA-seq. The single oocyte, zygote, and Eyal-giladi and Kochav stage X (EGK.X) blastoderm were obtained from one hen simultaneously. Samples was collected from three hens. Single embryo was sequenced as one replicate, and each stage consists of triplicated embryos from three hens, respectively. C) The representative stages of chicken early embryos used for RNA-seq. Dorsal views of whole embryos from the oocyte to EGK.X are shown. A germinal vesicle oocyte in the ovary and fertilized zygote in the magnum without cleavage were obtained. The intrauterine embryos were obtained 5.5 (EGK.I), 8.5 (EGK.III), 15.5 (EGK.VI), and 20.5 (EGK.VIII) hours after fertilization. The EGK.X embryo was obtained after oviposition. Scale bar, 1000 µm.

Recently, the Bird10K project was initiated because of the intermediate position of birds in the comparative biology of vertebrates and their broad utility for diverse research. This project used the genome sequences of 48 species of birds to construct a phylogenetic hierarchy of avian species and examine the comparative genomics of flight and functional adaptations [8–10]. However, no transcriptomic approach to early bird embryos has been performed. Here, we present whole transcriptome sequencing of bulked pre-oviposited chicken embryos, including oocyte, zygote, and intrauterine embryos from Eyal-giladi and Kochav stage I (EGK.I) to EGK.X (Fig. 1A). Furthermore, a single oocyte, zygote, and EGK.X blastoderm from one hen were sequenced (Fig.1B) and compared with the results for bulked embryos. Based on the whole transcriptome of early chicken embryos, we mapped our sequencing reads on the two most recent chicken (*Gallus gallus*) genome references, Galgal4 and Galgal5, and examined the differences in gene expression between the two builds with or without long intergenic non-coding RNA (lincRNA) annotations.

## Data Description

### Collection of bulked early chicken embryos

In the chicken, the initial 25 hours of embryonic development from fertilization to oviposition progresses through the oviduct. The mature oocyte on top of the yellow yolk is ovulated into the infundibulum 30 min after oviposition. Then, fertilization occurs and the zygote passes through the magnum without any morphological changes in the embryo. According to the well-defined criteria of Eyal-Giladi and Kochav [5, 6], the first cleavage is observed 5 hours after fertilization in the shell gland and has been designated EGK.I. Beginning with this event, the pre-ovipositional development of birds is divided into 10 stages, including the cleavage (EGK.I to EGK.VI) and area pellucida formation (EGK.VII to EGK.X) periods. During the cleavage stages, rapid cellularization and an increase in layers lead to formation of a multilayered blastula by EGK.VI. In the second half of intrauterine development, the first morphological segregation, including the area pellucida and area opaca regions, occurs with anterior–posterior axis formation and layer reduction. Finally, a thinner, longer, bilayered blastoderm is established at EGK.X. Based on the morphological dynamics that occur during intrauterine development, we chose the following critical representative stages to analyze: the oocyte, the zygote, EGK.I, EGK.III, EGK.VI, EGK.VIII, and EGK.X (Fig.1A).

**Figure 2:**
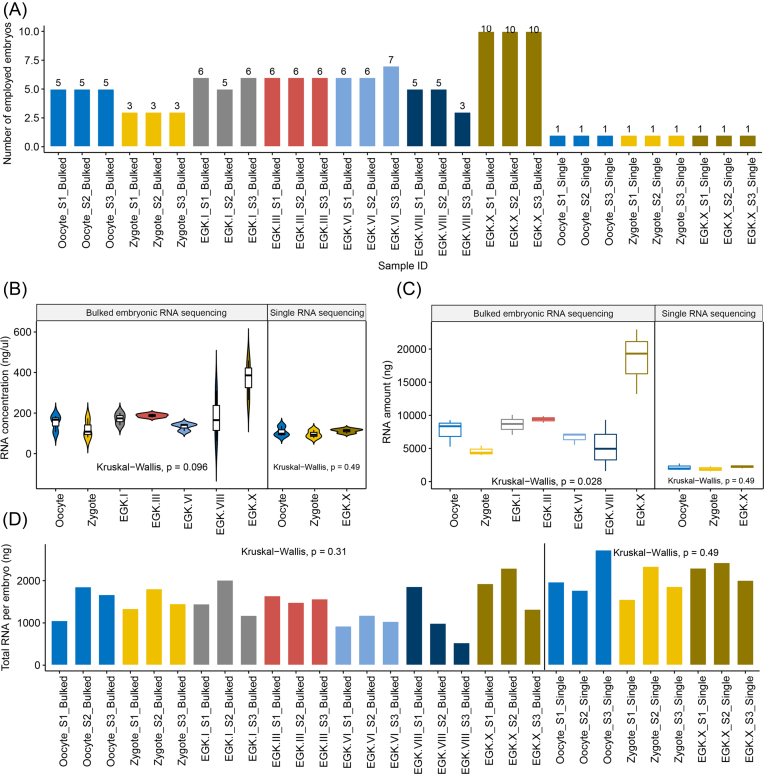
Collection of bulked and single embryos during early chicken development. A) The number of embryos in each sample. B) The RNA concentration. C) The total amount of RNA for each stage used in RNA-seq. D) The estimated total RNA per embryo in the bulked samples and the total amount of RNA in a single embryo. The RNA concentration, amount of RNA, and total RNA per embryo did not differ significantly among the groups (Kruskal–Wallis test, *P* > 0.05).

The egg-laying times of white leghorn (WL) hens were recorded, and intrauterine eggs from EGK.I−VIII were harvested using an abdominal massage technique [11]. Briefly, the abdomen was pushed gently until the shell gland was exposed; the surface of the shell gland expands when an egg is present for egg shell formation. After expansion of the shell gland surface, massaging was used to move the egg gently toward the cloaca until the intrauterine egg was released. EGK.X blastoderms were collected from WL hens after oviposition. To collect oocytes and zygotes, WL hens were sacrificed, and the follicles were collected. Zygote embryos located in the magnum and showing no cleavage were collected within 1 hour post-fertilization according to the recorded egg-laying times. All embryos were classified according to morphological criteria (Fig.1C). All stages were prepared in triplicate and each replicate contained 3 to 7 embryos, while there were 10 embryos per replicate of the post-oviposited EGK.X blastoderm (Fig.2A). Shortly after collection, the embryos were separated from the egg using sterilized paper, and the shell membrane and albumen were detached from the yolk. A piece of square filter paper (Whatman, Maidstone, UK) with a hole in the center was placed over the germinal disc. After cutting around the paper containing the embryo, it was gently turned over and transferred to saline to remove the yolk and vitelline membrane and allow embryo collection. Total RNA was isolated from early embryos using TRIzol reagent (Invitrogen, Carlsbad, CA, USA). The quality and quantity of the extracted total RNA were determined using the Trinean DropSense96 system (Trinean, Gentbrugge, Belgium), a RiboGreen kit (Invitrogen), and an Agilent 2100 Bioanalyzer (Agilent Technologies, Santa Clara, CA, USA). We assessed the rRNA ratio (28s: 18 s) and RNA integrity number (RIN) of bulked embryos (Table S1: Additional file 1). We observed a lower rRNA ratio from zygote to EGK.VIII stage, because of the low levels of 28 s rRNA before maternal-to-zygotic transition (MZT) during early development [12, 13]. The average concentration and amount of total RNA in the early stages was 157.7 ng/μL and 7,026.2ng, respectively, with the exception of EGK.X, which contained 368.9 ng/μL and 18,495.8ng due to the larger number of embryos pooled (Fig.2B and C). Based on the amount of total RNA and the number of embryos in each sample, we estimated the total amount of RNA per embryo in each stage. On average, the early chicken embryos contained 1,457ng of total RNA (Fig.2D).

### Collection of a single oocyte, zygote, and EGK.X blastoderm from one hen

In accordance with the estimated amount of total RNA per embryo, a single RNA-rich embryo could be used to perform RNA-sequencing (RNA-seq) without an amplification technique. In this way, probable sequencing errors due to library amplification from low-input RNA can be avoided. Furthermore, the deviation of transcriptomes among early embryos at the same stage can be examined. Chicken physiology allows a single oocyte, zygote, and EGK.X blastoderm to be collected from one hen at the same time, which minimizes any individual variation and maternal effects (Fig.1B). On the day when single embryos were acquired, a single EGK.X blastoderm was collected and the time was recorded. Within 1 hour post-fertilization according to the recorded egg-laying times, a WL hen was sacrificed and a single oocyte and zygote were simultaneously collected. All stages were prepared in triplicate (Fig.2A). The subsequent steps, including embryo separation and total RNA isolation and quantification, were the same as for the pooled embryos. RIN of single embryos were comparable to bulked embryos (Table S1: Additional file 1). With the single-embryo approach, the RNA concentration was 105.3 ng/μL and the amount of total RNA averaged 2,123.5ng (Fig.2B and C). The total amount of RNA for a single embryo was higher and more constant among the different stages than with the bulked embryo collection (Fig.2D).

### Library preparation and whole transcriptome sequencing

Total RNA was used to construct cDNA libraries using the TruSeq Stranded Total RNA Sample Preparation kit with Ribo-Zero Gold (Illumina, San Diego, CA, USA). The resulting average size of the cDNA libraries was approximately 530 bp. The resulting libraries were subjected to transcriptome analysis using the Illumina NextSeq 500 platform to produce 150 bp paired-end reads.

### Summary statistics of preprocessing for RNA-seq data

Thirty RNA-seq samples were used in the preprocessing step for the quantification of gene expression in the early developmental stages in the chicken. First, adapter sequences and poor-quality reads were removed from the raw paired-end sequenced files using Trimmomatic ver. 0.33 with the “-phred33 and ILLUMINACLIP:/home/Program/Trimmomatic-0.32/adapters/TruSeq3-PE-2.fa:2:30:10 MINLEN:75 option” [14]. The quality of the clean reads, including minimum read length >75 bp and Phred score >30, was verified using FastQC ver. 0.11.2 [15]. On average, 58,930,612 (96.75%) and 39,969,608 (86.16%) paired-end reads remained after the quality-control step for bulked and single-embryo sequencing, respectively (Table 1).

**Table 1:** Summary statistics of the RNA-seq processing

Bulked embryonic sequencing								
Samples	QC passed reads	QC passing rate	Deduplicated percentage (R1)	Deduplicated percentage (R2)	Uniquely mapped ratio (Galgal4)	Uniquely mapped ratio (Galgal5)	Mapping rates (Galgal4)	Mapping rates (Galgal5)
Oocyte_S1_Bulked	56, 024 ,575	94.81%	37.83%	44.47%	83.82%	99.56%	82.73%	84.32%
Oocyte_S2_Bulked	56, 043, 780	94.14%	35.35%	42.25%	81.27%	99.34%	82.77%	79.54%
Oocyte_S3_Bulked	59 ,498, 675	95.54%	35.84%	43.31%	84.03%	99.58%	82.16%	82.39%
Zygote_S1_Bulked	53, 378, 148	96.74%	30.17%	37.65%	84.77%	99.74%	82.43%	85.89%
Zygote_S2_Bulked	53 ,999, 584	96.77%	26.44%	35.21%	84.20%	99.73%	82.19%	79.86%
Zygote_S3_Bulked	50, 027, 929	98.02%	25.13%	39.78%	86.17%	99.76%	80.90%	87.58%
EGK.I_S1_Bulked	56 ,909 ,314	97.36%	27.88%	39.30%	86.28%	86.01%	74.55%	70.70%
EGK.I_S2_Bulked	61, 447, 014	97.94%	21.97%	36.34%	87.13%	86.86%	73.24%	68.64%
EGK.I_S3_Bulked	50 ,188 ,847	96.80%	28.24%	37.31%	85.33%	84.67%	81.34%	77.01%
EGK.III_S1_Bulked	60 ,876 ,681	97.30%	25.31%	36.29%	85.09%	86.05%	76.06%	69.37%
EGK.III_S2_Bulked	56, 357 ,690	97.90%	25.78%	38.44%	86.55%	86.28%	75.20%	70.47%
EGK.III_S3_Bulked	45, 715, 485	98.02%	28.17%	41.24%	86.72%	86.32%	75.30%	70.38%
EGK.VI_S1_Bulked	62 ,075, 038	97.53%	27.00%	42.86%	86.62%	86.77%	71.14%	63.68%
EGK.VI_S2_Bulked	65, 223 ,164	97.77%	23.36%	34.43%	85.85%	85.61%	80.95%	72.89%
EGK.VI_S3_Bulked	49 ,604, 292	98.16%	27.31%	41.37%	86.86%	86.44%	75.12%	69.22%
EGK.VIII_S1_Bulked	67, 401 ,388	97.35%	36.30%	50.09%	87.21%	86.30%	70.10%	67.32%
EGK.VIII_S2_Bulked	56,396 ,268	96.82%	35.34%	51.04%	87.61%	87.25%	66.53%	60.37%
EGK.VIII_S3_Bulked	71, 309, 063	97.44%	33.44%	49.01%	86.81%	85.62%	70.68%	70.70%
EGK.X_S1_Bulked	67 ,730 ,502	95.70%	41.09%	52.23%	86.37%	85.54%	72.24%	69.29%
EGK.X_S2_Bulked	74 ,109 ,500	95.02%	42.83%	54.66%	86.60%	85.48%	70.64%	69.62%
EGK.X_S3_Bulked	63, 225, 919	94.65%	42.42%	54.85%	86.86%	85.82%	71.13%	69.51%
**Average**	58, 930, 612.19	0.967514286	31.30%	42.96%	85.82%	89.94%	76.06%	73.27%
Single embryonic or cell sequencing								
Oocyte_S1_SingleCell	23, 558, 381	86.61%	42.82%	45.68%	79.90%	78.06%	86.28%	86.67%
Oocyte_S2_SingleCell	53 ,963, 445	84.75%	54.54%	58.84%	81.28%	79.71%	85.95%	85.86%
Oocyte_S3_SingleCell	24, 660, 386	84.95%	52.01%	56.95%	81.79%	79.99%	84.95%	84.33%
Zygote_S1_SingleEmbryo	31, 742 ,857	87.17%	27.95%	32.85%	81.75%	80.66%	84.32%	84.40%
Zygote_S2_SingleEmbryo	91 ,033, 778	85.72%	37.12%	42.18%	81.62%	80.14%	76.59%	76.15%
Zygote_S3_SingleEmbryo	27, 687, 195	87.60%	36.35%	41.48%	81.57%	80.07%	86.02%	85.96%
EGK.X_S1_SingleEmbryo	30, 914, 824	86.41%	47.58%	51.56%	85.27%	82.92%	83.67%	83.16%
EGK.X_S2_SingleEmbryo	47, 159, 061	86.29%	53.42%	58.46%	82.40%	80.75%	88.38%	89.10%
EGK.X_S3_SingleEmbryo	29, 006, 546	85.94%	51.19%	55.97%	82.20%	80.59%	83.57%	82.86%
**Average**	39, 969, 608.11	0.8616	44.78%	49.33%	81.98%	80.32%	84.41%	84.27%

The clean reads were mapped into the two builds of the Galgal4 and Galgal5 reference genomes, which were obtained from the Ensembl database. The Galgal4 build was the so-called golden standard reference chicken genome at the end of 2015, and many studies have used this build. In December 2016, a new genome build, Galgal5 (Ref Seq assembly accession: GCA_0 00002315.3), and an improved gene model were established using advanced sequencing techniques. One of the features of Galgal5 compared with Galgal4 is the different read length used when the gene model was established. This change improved inaccurate gene annotations, especially the structure of isoforms, in existing short-read based gene models through an isoform sequencing technique using the Pacific Biosciences (PacBio) long reads. Furthermore, PacBio long-read sequencing technology makes it possible to establish lincRNAs, which is important in developmental biology [16]. Given that our data were not only an early developmental sample of a chicken but also a sample of all types of RNAs, this must be considered when quantifying gene expression levels in the RNA-seq pipeline. Therefore, we decided to quantify the expression level of the entire transcriptome using the two versions of the genome builds and then compare the results in order to examine the differences. In the alignment step, HISAT2 ver. 2.0.0 [17] was used with “–rna-strandness RF –x [File name of Galgal4 or Galgal5 reference] -1 [File name of left lead] -2 [File name of right read] 2> [Sample name].log.” As a result, an average of 76.07% and 73.27% mapping rates were observed in Galgal4 and Galgal5, respectively, in the 21 bulked embryo samples and 84.41% and 84.28% were observed in the nine single embryo or cell samples (Table 1). For Galgal4 and Galgal5, the average observed difference in the mapping rates between the bulked and single embryo samples was 8.35% and 11%, respectively. We suspected that this difference in mapping rates was caused by the individual gene expression diversity. Upon examining the duplication rate of the generated read, a higher duplication rate was observed in single cell and/or embryonic RNA-seq, which is evidence that the individual gene expression diversity is lower in single embryonic samples (Table 1). Since transcriptome data generated using single embryo sequencing technology contains only its own gene expression for a single entity, it is assumed that the mapping rate is increased by alleviating the heterogeneity problem derived from various individuals. We also observed small differences in the average mapping rates in two genome builds; 2.79% and 0.14% decrease, respectively, for the bulked and single embryo samples in Galgal5 compared to Galgal4, which implies that there are few differences between the two genome builds at the DNA level, but more impact on bulked embryos. Following the alignment step using the two versions of the genome builds, alignment files (.SAM files) were converted into binary alignment files (.BAM) using SAMtools ver. 1.4.1 [18]. Based on the alignment files, the gene expression levels (number of mapped reads) were quantified using HTSeq-count [19] with the following option: “python -m HTSeq.scripts.count -f bam –stranded = reverse [File name of bam file] [File name of annotation (.GTF file)] > [Output file name],” with the Ensembl gene annotation files corresponding to the genome builds (Ensembl release 85 for Galgal4 and 86 for Galgal5). As a result, the number of mapped reads was quantified in each pipeline, and 17,108 and 26,102 genes were annotated in the Galgal4 and Galgal5 genome builds, respectively.

### Comparison of the gene expression patterns between Galgal4 and Galgal5 in chicken early embryo samples

**Figure 3:**
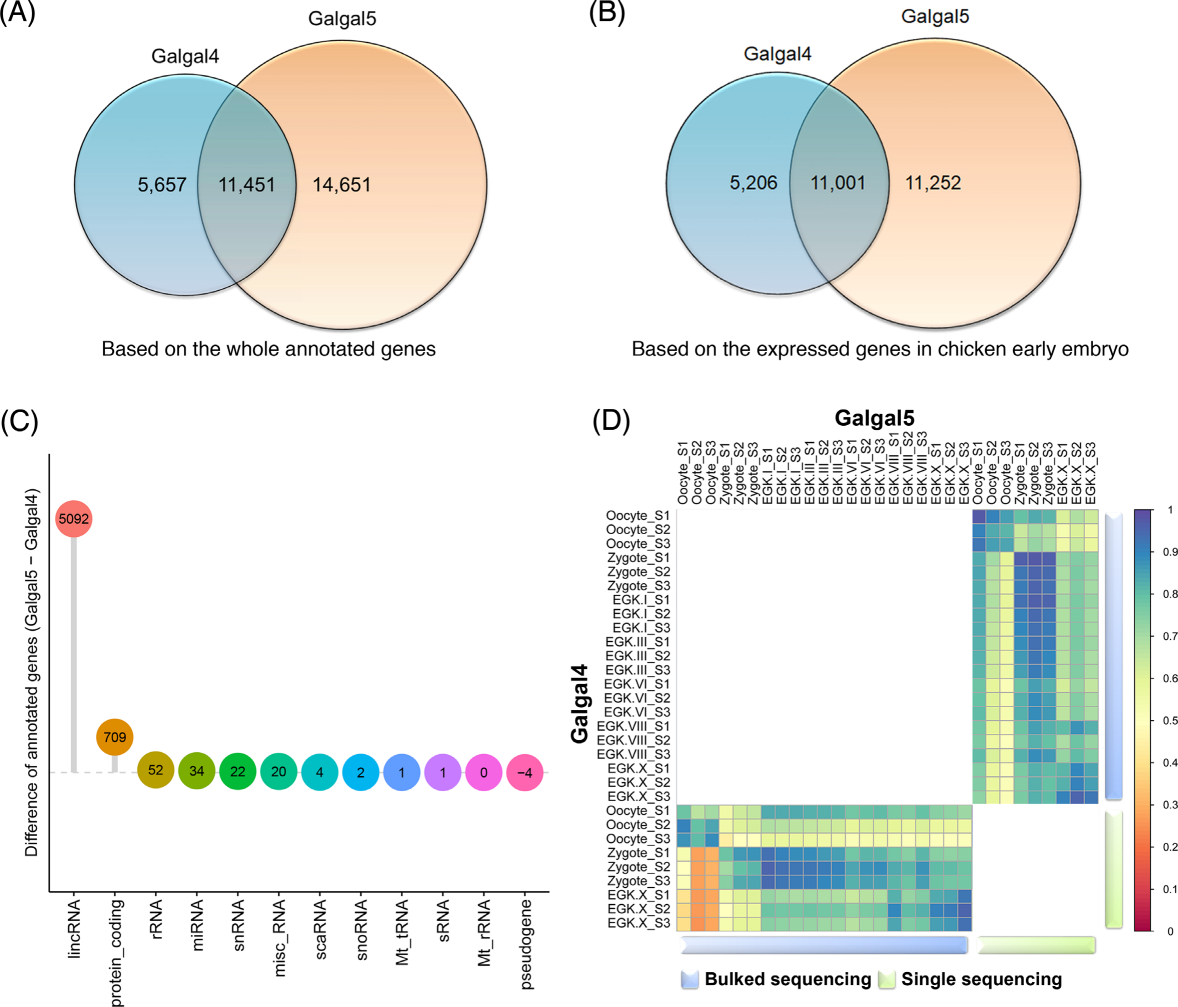
Comparison of two builds of gene annotation for the early chicken embryo samples. A) Using the Ensembl annotation with the two genome builds, annotated genes were compared based on the Ensembl ID. As a result, 5,657 and 14,651 Ensembl IDs were identified in Galgal4 and Galgal5, respectively, while 11,451 Ensembl IDs are common to the two different annotations. B) Based on the expressed genes at any stage of chicken early embryos, the gene lists were compared between Galgal4 and Galgal5. C) Investigation of the change in annotated genes in Galgal5 among genes expressed in early chicken embryos. As a result, a large number of lincRNAs was added as new features in Galgal5. D) A correlation analysis of the total gene expression based on 11,001 common annotated genes shared between Galgal4 and Galgal5.

Based on the mapped-count matrix of the genome builds and the Ensembl annotation, we systematically investigated how many and which types of genes differed between the two genome builds. First, we found that many genes were differentially annotated in each build in terms of their Ensembl IDs (Fig. 3A). Of the 17,108 and 26,102 annotated genes in Galgal4 and Galgal5, respectively, only 11,451 Ensembl IDs were shared by both annotations, while 5,657 and 14,651 Ensembl IDs were annotated only in the respective builds. Next, we compared the two genome builds based on the genes actually expressed in the early embryo samples of chickens. For this comparison, we filtered out genes with no mapped counts across all 30 RNA-seq samples. As a result, 901 and 3,849 genes were filtered out in the raw gene annotations of Galgal4 and Galgal5, respectively (i.e., 16,207 and 22,253 genes remained). Because the same pattern of results was observed when validated with the filtered Ensembl IDs (Fig.3B), we then examined which RNA type produced the difference between Galgal4 and Galgal5. As a result, many lincRNAs and protein-coding genes were newly identified in Galgal5 (Table 2) and confirmed to be expressed in early chicken embryos (Fig. 3C). With the development of sequencing technology, lincRNA has been added to more than 5,166 new genes, and it has been confirmed that it is actually expressed in our data based on the mapped reads. Unlike lincRNA, which was unilaterally added to Galgal5, there were many changes in protein-coding genes (Table 2). A total of 4,892 protein-coding genes were discarded, while 5,613 were added to the new version of the gene annotation (based on the Ensembl ID matching). Since there is still a lack of empirical evidence and practical discussion of the validity of both gene models, it is impossible to determine which genome build is correct for quantifying gene expression in our study. However, we expect to contribute to further studies by providing the entire transcript expression metrics for early embryos of chickens in both builds. Finally, correlations between the 30 samples were examined based on the quantified expression of 11,001 genes common to the gene annotations of these two builds (Fig.3D). Based on bulked embryo sequencing, high correlations (≥0.9) were observed between Galgal4 and Galgal5, except for the oocyte and zygote. In comparison, single embryo and/or cell sequencing showed the high correlation between Galgal4 and Galgal5, including the oocyte and zygote. This demonstrates the excellent reproducibility of the data produced based on the single experimental subject. Most of the embryonic transcriptome data generated to date have involved pooling problems, and we expect to be able to perform more sophisticated downstream analysis using single embryo and/or cell sequencing, which is now possible due to technological developments.

**Table 2:** Comparison of Galgal4 and Galgal5 gene annotations

RNAs	Annotated in Galgal4 only	Commonly annotated	Annotated in Galgal5 only
lincRNA	0	0	5,166
miRNA	204	487	253
misc_RNA	15	71	43
Mt_rRNA	2	0	2
Mt_tRNA	10	0	14
protein_coding	4,892	10, 213	5,613
pseudogene	29	10	25
rRNA	6	8	58
scaRNA	0	0	4
snoRNA	41	172	44
snRNA	7	40	30
Total	5,206	11, 001	11, 252

### Comparison of bulked embryo sequencing and single embryo and/or cell sequencing with chicken early embryos

**Figure 4:**
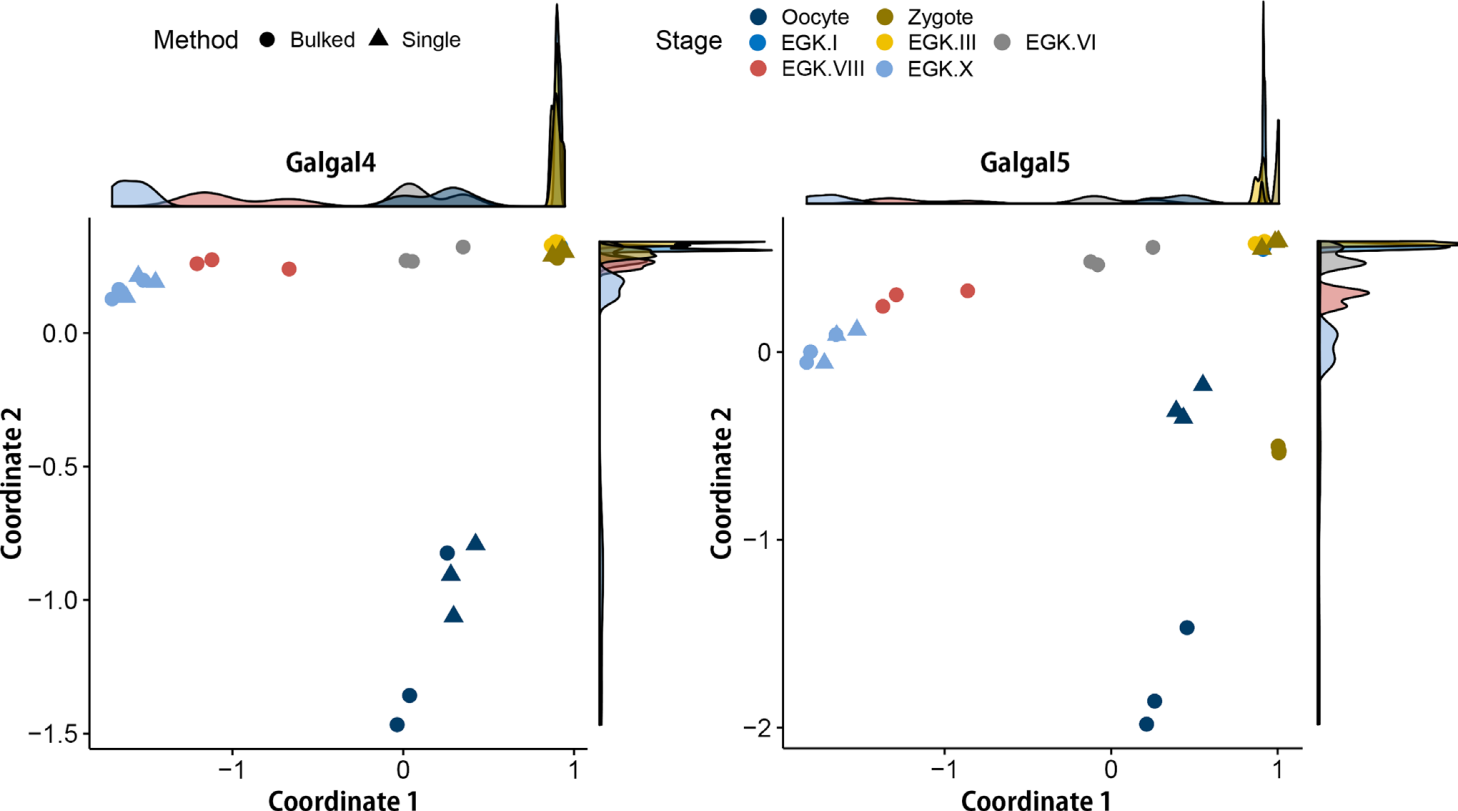
Multidimensional scaling plots based on all annotated genes in Galgal4 and Galgal5. The gene expression patterns of early chicken embryos quantified based on Galgal4 were clearly differentiated by developmental stage regardless of the sequencing technique used. In comparison, there was a difference between the bulked and single embryo sequencing techniques in the oocyte and zygote in Galgal5. The first dimension (Coordinate 1) is the progression of developmental stages in a negative direction during intrauterine development, and the second dimension (Coordinate 2) is the difference between oocyte and fertilized embryos from zygote to EGK.X.

To investigate the differences between the two technologies more systematically, multidimensional scaling (MDS)analysis was performed using information from 30 RNA-seq samples in two gene expression matrixes: Galgal4 and Galgal5. All of the samples in both gene expression matrixes clearly clustered according to their developmental stage, except for the zygote, EGK.I, and EGK.III (Fig.4). This means that although there are morphological differences, there is no transcriptome change during the early embryonic development of the chicken for a specific time after zygotic gene activation. In fact, the time from the zygote to EGK.III is also very short. While most of the patterns seem to be concordant between Galgal4 and Galgal5, distinct differences were observed between the bulked and single embryo RNA-seq techniques for the oocyte and zygote samples based on the Galgal5 gene expression matrix. However, no difference was detected between the two techniques for the EGK.X samples, which is presumably due to the difference between the bulked and single cells because we performed single embryo RNA-seq for the oocyte, zygote, and EGK.X stages. The RNA samples from the oocyte and zygote were derived from a single cell, whereas those from EGK.X were derived from bulked cells. As we have already examined the difference in gene annotation between Galgal4 and Galgal5, more than 10,000 genes have been changed, which includes both protein-coding genes and lincRNAs. Of these changes, 5,166 newly added lincRNAs may be a major factor causing this difference because lincRNA plays an important role in the zygote as an epigenetic marker in both humans and mice, which have been subjected to lincRNA annotation and early embryonic transcription studies. Furthermore, epigenetic markers are very sensitive, exhibiting subject- or cell-specific characteristics. Therefore, our RNA-seq data based on the single embryo and cell technique for oocytes and zygotes are more accurate than ordinary RNA-seq data because they eliminate epigenetic and genetic pooling effects. For example, bulked zygote samples were separated from the cluster of EGK.I and EGK.III samples in a MDS analysis based on the Galgal5 gene matrix, whereas there was no difference in the Galga4 gene expression matrix (Fig.4, right panel). This shows that quantifying gene expression using the standard RNA-seq pooled embryo sequencing technique could be affected by the individual gene expression diversity and the difference of gene annotations.

In summary, we produced the first whole transcriptome sequences of pre-oviposited early chicken embryos based on standard RNA-seq and single embryo sequencing techniques. We then quantified and compared gene expression using the standard gene annotation used for the chicken and a new chicken gene annotation based on the advanced long-read sequencing technique. As a result, we not only demonstrated the accuracy of RNA-seq data based on single embryo or cell sequencing but also successfully quantified 5,166 lincRNAs in the new chicken gene model for the pre-oviposited early chicken embryo. We expect that the transcriptome sequences of pre-oviposited early chicken embryos will fill the gap in comparative developmental and evolutionary studies of vertebrates as a valuable resources and provide comprehensive knowledge of early avian embryogenesis. Furthermore, the oocyte and early chicken embryos express numerous types of RNA, including mRNA and lincRNA, so our dataset should help to establish novel transcript and gene annotations for the chicken reference genome. Our large dataset should also be useful for future studies of avian and comparative genomics because the data were generated using the latest sequencing platform and whole transcriptome sequencing enabling the characterization of all RNA transcripts, including primary transcripts, regardless of polyadenylation.

## Supplementary Material

GIGA-D-17-00277_Original_Submission.pdf

GIGA-D-17-00277_Revision_1.pdf

Response_to_Reviewer_Comments_Original_Submission.pdf

Reviewer_1_Report_(Original_Submission) -- Laure Fresard 10 Dec 2017 Reviewed

Reviewer_2_Original_Submission_Attachment_Enke_review-GIGA-D-17-00277.pdf

Reviewer_2_Report_(Original_Submission) -- Raymond Enke 12 Dec 2017 Reviewed

Reviewer_2_Report_(Revision_1) -- Raymond Enke 15 Feb 2018 Reviewed

Table S1

## Availability of supporting data

The bulked and single embryo RNA-seq data have been deposited in the National Center for Biotechnology Information GEO database (GSE86592 and GSE100798, respectively). Supporting data including preprocessed gene expression levels are also available in the GigaScience database, GigaDB [20].

## Additional file

Table S1: rRNA ratio during pre-ovipositional development and RNA integrity number (RIN) of the RNA-seq samples.

## Abbreviations

EGK, Eyal-giladi and Kochav; lincRNA, long intergenic non-coding RNA; MDS, multidimensional scaling; PacBio, Pacific Biosciences; RIN: RNA integrity number; RNA-seq, RNA-sequencing; WL, white leghorn.

## Experimental animals and animal care

The care and experimental use of chickens were approved by the Institute of Laboratory Animal Resources, Seoul National University (SNU-150827-1). Chickens were maintained according to a standard management program at the University Animal Farm, Seoul National University, Korea. The procedures for animal management, reproduction, and embryo manipulation adhered to the standard operating protocols of our laboratory.
