# Supplementary material for: The first whole transcriptomic exploration of pre-oviposited early chicken embryos using single and bulked embryonic RNA-sequencing
Source: Gigascience. 2018 Mar 24;7(4):giy030. doi: 10.1093/gigascience/giy030 (PMC5893961; doi:10.1093/gigascience/giy030)

GigaScience

The first whole transcriptomic exploration of pre-oviposited early chicken embryos using single and bulked embryonic RNA-sequencing

--Manuscript Draft--

**Manuscript Number:**	GIGA-D-17-00277
**Full Title:**	The first whole transcriptomic exploration of pre-oviposited early chicken embryos using single and bulked embryonic RNA-sequencing
**Article Type:**	Data Note
**Funding Information:**	National Research Foundation of Korea (2015R1A3A2033826) — Prof. Jae Yong Han
**Abstract:**	Background<br>The chicken is a valuable model organism, especially in evolutionary and embryology research because its embryonic development occurs in the egg. However, despite its scientific importance, no transcriptome data have been generated for deciphering the early developmental stages of the chicken because of practical and technical constraints accessing pre-oviposited embryos.<br>Findings<br>Here, we determine the entire transcriptome of pre-oviposited avian embryos, including oocyte, zygote, and intrauterine embryos from Eyal-giladi and Kochav stage I (EGK.I) to EGK.X collected using a non-invasive approach for the first time. We also compare RNA-sequencing data obtained using bulked embryo sequencing and single embryo/cell sequencing technique. The raw sequencing data were pre-processed with two different genome builds, Galgal4 and Galgal5, and the expression of 17,108 and 26,102 genes was quantified in the respective builds. There were some differences between the two techniques, as well as between the two genome builds, and these were affected by the emergence of long intergenic non-coding RNA annotations.<br>Conclusion<br>The first transcriptome datasets of pre-oviposited early chicken embryos based on bulked and single embryo sequencing techniques will serve as a valuable resource for investigating early avian embryogenesis, for comparative studies among vertebrates, and for novel gene annotation in the chicken genome.
**Corresponding Author:**	Jae Yong Han, Ph.D.<br>Seoul National University<br>Gwanak-gu, Seoul KOREA, REPUBLIC OF
**Corresponding Author Secondary Information:**	
**Corresponding Author's Institution:**	Seoul National University
**Corresponding Author's Secondary Institution:**	
**First Author:**	Young Sun Hwang
**First Author Secondary Information:**	
**Order of Authors:**	Young Sun Hwang
	Minseok Seo
	Hee Jung Choi
	Sang Kyung Kim
	Heebal Kim
	Jae Yong Han, Ph.D.
**Order of Authors Secondary Information:**	
**Opposed Reviewers:**	
**Additional Information:**	

Powered by Editorial Manager® and ProduXion Manager® from Aries Systems Corporation

Question	Response
Are you submitting this manuscript to a special series or article collection?	No
**Experimental design and statistics**<br><br>Full details of the experimental design and statistical methods used should be given in the Methods section, as detailed in our Minimum Standards Reporting Checklist. Information essential to interpreting the data presented should be made available in the figure legends.<br><br>Have you included all the information requested in your manuscript?	Yes
**Resources**<br><br>A description of all resources used, including antibodies, cell lines, animals and software tools, with enough information to allow them to be uniquely identified, should be included in the Methods section. Authors are strongly encouraged to cite Research Resource Identifiers (RRIDs) for antibodies, model organisms and tools, where possible.<br><br>Have you included the information requested as detailed in our Minimum Standards Reporting Checklist?	Yes
**Availability of data and materials**<br><br>All datasets and code on which the conclusions of the paper rely must be either included in your submission or deposited in publicly available repositories (where available and ethically appropriate), referencing such data using a unique identifier in the references and in the “Availability of Data and Materials” section of your manuscript.<br><br>Have you have met the above requirement as detailed in our Minimum Standards Reporting Checklist?	Yes

Manuscript
Click here to download Manuscript 171020_Manuscript_Gigascience.docx

The first whole transcriptomic exploration of pre-oviposited early chicken embryos using single and bulked embryonic RNA-sequencing

Young Sun Hwang[1,†], Minseok Seo[2,3,†], Hee Jung Choi[1], Sang Kyung Kim[1], Heebal Kim[1,2,4], and Jae Yong Han[1,4,*]

[†]These authors contributed equally to this work

*Corresponding Author
Jae Yong Han, Ph.D.
Seoul National University, 1 Gwanak-ro, Gwanak-gu, Seoul 08826, Korea
Phone: +82-2-880-4810
FAX: +82-2-874-4811
E-mail: jaehan@snu.ac.kr

Abstract

Background

The chicken is a valuable model organism, especially in evolutionary and embryology research because its embryonic development occurs in the egg. However, despite its scientific importance, no transcriptome data have been generated for deciphering the early developmental stages of the chicken because of practical and technical constraints accessing pre-oviposited embryos.

Findings

Here, we determine the entire transcriptome of pre-oviposited avian embryos, including oocyte, zygote, and intrauterine embryos from Eyal-giladi and Kochav stage I (EGK.I) to EGK.X collected using a non-invasive approach for the first time. We also compare RNA-sequencing data obtained using bulked embryo sequencing and single embryo/cell sequencing technique. The raw sequencing data were pre-processed with two different genome builds, Galgal4 and Galgal5, and the expression of 17,108 and 26,102 genes was quantified in the respective builds. There were some differences between the two techniques, as well as between the two genome builds, and these were affected by the emergence of long intergenic non-coding RNA annotations.

Conclusion

The first transcriptome datasets of pre-oviposited early chicken embryos based on bulked and single embryo sequencing techniques will serve as a valuable resource for investigating early avian embryogenesis, for comparative studies among vertebrates, and for novel gene annotation in the chicken genome.

Keywords

RNA-seq - Single embryonic sequencing - Single cell sequencing - Early embryo - Chicken

Background

Avian species are valuable animal models in many research areas, especially in embryology, because the avian embryo develops in an egg before hatching. This is an excellent *in vitro*-like *in vivo* system that has allowed extensive research of the developmental events during embryogenesis. Previous studies have examined primitive streak formation and gastrulation after oviposition in avian species [1–4]. Nevertheless, despite the importance of the initial events in avian embryogenesis before oviposition, only a few morphological studies have examined pre-oviposited embryos because of practical difficulties accessing the embryos [5–7]. The temporal regulation of gene expression during the pre-oviposited stages is important for understanding early embryonic development.

Recently, the Bird10K project was initiated because of the intermediate position of birds in the comparative biology of vertebrates and their broad utility for diverse research. This project used the genome sequences of 48 species of birds to construct a phylogenetic hierarchy of avian species and examine the comparative genomics of flight and functional adaptations [8–10]. However, no transcriptomic approach to early bird embryos has been performed. Here, we present whole transcriptome sequencing of pre-oviposited chicken embryos, including oocyte, zygote, and intrauterine embryos from Eyal-giladi and Kochav stage I (EGK.I) to EGK.X (Fig. 1a). Furthermore, a single oocyte, zygote, and EGK.X blastoderm from one hen were sequenced (Fig. 1b) and compared with the results for bulked embryos. Based on the whole transcriptome of early chicken embryos, we mapped our sequencing reads on the two most recent chicken (*Gallus gallus*) genome references, Galgal4 and Galgal5, and examined the differences in gene expression between the two builds with or without long intergenic non-coding RNA (lincRNA) annotations.

Data description

Collection of bulked early chicken embryos

In the chicken, the initial 25 h of embryonic development from fertilization to oviposition progresses through the oviduct. The mature oocyte on top of the yellow yolk is ovulated into the infundibulum 30 min after oviposition. Then, fertilization occurs and the zygote passes through the magnum without any morphological changes in the embryo. According to the well-defined criteria of Eyal-Giladi and Kochav [5, 6], the first cleavage is observed 5 h after fertilization in the shell gland and has been designated EGK.I. Beginning with this event, the pre-ovipositional development of birds is divided into 10 stages, including the cleavage (EGK.I to EGK.VI) and area pellucida formation (EGK.VII to EGK.X) periods. During the cleavage stages, rapid cellularization and an increase in layers lead to formation of a multi-layered blastula by EGK.VI. In the second half of intrauterine development, the first morphological segregation, including the area pellucida and area opaca regions, occurs with anterior–posterior axis formation and layer reduction. Finally, a thinner, longer, bi-layered blastoderm is established at EGK.X. Based on the morphological dynamics that occur during intrauterine development, we chose critical representative stages to analyze: EGK.I, EGK.III, EGK.VI, EGK.VIII, EGK.X, the oocyte, and the zygote (Fig. 1a).

The egg-laying times of white leghorn (WL) hens were recorded, and intrauterine eggs from EGK.I−VIII were harvested using an abdominal massage technique [11]. Briefly, the abdomen was pushed gently until the shell gland was exposed; the surface of the shell gland expands when an egg is present for egg shell formation. After expansion of the shell gland surface, massaging was used to move the egg gently towards the cloaca until the intrauterine egg was released. EGK.X blastoderms were collected from WL hens after oviposition. To collect oocytes and zygotes, WL hens were sacrificed and the follicles were collected. Zygote embryos located in the magnum and showing no cleavage were collected within 1 h post-fertilization according to the recorded egg-laying times. All embryos were classified according to morphological criteria (Fig. 1c). All stages were prepared in triplicate and each replicate contained three to seven embryos, while there were ten embryos per

replicate of the post-oviposited EGK.X blastoderm (Fig. 2a). Shortly after collection, the embryos were separated from the egg using sterilized paper, and the shell membrane and albumen were detached from the yolk. A piece of square filter paper (Whatman, Maidstone, UK) with a hole in the center was placed over the germinal disc. After cutting around the paper containing the embryo, it was gently turned over and transferred to saline to remove the yolk and vitelline membrane and allow embryo collection. Total RNA was isolated from early embryos using TRIzol reagent (Invitrogen, Carlsbad, CA, USA). The quality and quantity of the extracted total RNA were determined using the Trinean DropSense96 system (Trinean, Gentbrugge, Belgium), a RiboGreen kit (Invitrogen), and an Agilent 2100 Bioanalyzer (Agilent Technologies, Santa Clara, CA, USA). The average concentration and amount of total RNA in the early stages was 157.7 ng/μL and 7,026.2 ng, respectively, with the exception of EGK.X, which contained 368.9 ng/μL and 18,495.8 ng due to the larger number of embryos pooled (Fig. 2b, c). Based on the amount of total RNA and the number of embryos in each sample, we estimated the total amount of RNA per embryo in each stage. On average, the early chicken embryos contained 1,457 ng of total RNA (Fig. 2d).

Collection of a single oocyte, zygote, and EGK.X blastoderm from one hen

In accordance with the estimated amount of total RNA per embryo, a single RNA-rich embryo could be used to perform RNA-sequencing (RNA-Seq) without an amplification technique. In this way, probable sequencing errors due to library amplification from low-input RNA can be avoided. Furthermore, the deviation of transcriptomes among early embryos at the same stage can be examined. Chicken physiology allows a single oocyte, zygote, and EGK.X blastoderm to be collected from one hen at the same time, which minimizes any individual variation and maternal effects (Fig. 1b). On the day when single embryos were acquired, a single EGK.X blastoderm was collected and the time was recorded. Within 1 h post-fertilization according to the recorded egg-laying times, a WL hen was sacrificed and a

single oocyte and zygote were simultaneously collected. All stages were prepared in triplicate (Fig. 2a). The subsequent steps, including embryo separation and total RNA isolation and quantification, were the same as for the pooled embryos. With the single-embryo approach, the RNA concentration was 105.3 ng/μL and the amount of total RNA averaged 2123.5 ng (Fig. 2b, c). The total amount of RNA for a single embryo was higher and more constant among the different stages than with the bulked embryo collection (Fig. 2d).

Library preparation and whole transcriptome sequencing

Total RNA was used to construct cDNA libraries using the TruSeq Stranded Total RNA Sample Preparation kit (Illumina, San Diego, CA, USA). The resulting libraries were subjected to transcriptome analysis using the Illumina NextSeq 500 platform to produce paired 150 base pair reads.

Summary statistics of pre-processing for RNA-seq data

Thirty RNA-seq samples were used in the pre-processing step for the quantification of gene expression in the early developmental stages in the chicken. First, adapter sequences and poor-quality reads were removed from the raw paired-end sequenced files using Trimmomatic ver. 0.33 [12]. The quality of the clean reads was verified using FastQC ver. 0.11.2 [13]. On average, 58,930,612 (96.75%) and 39,969,608 (86.16%) paired-end reads remained after the quality-control step for bulked and single-embryo sequencing, respectively (Table 1).

The clean reads were mapped into the two different builds of the Galgal4 and Galgal5 reference genomes, which were obtained from the Ensembl database. The Galgal4 build was the so-called “golden standard” reference chicken genome at the end of 2015, and many studies have employed this build. In December 2016, a new genome build, Galgal5

(Ref Seq assembly accession: GCA_000002315.3) and an improved gene model were established using advanced sequencing techniques. One of the features of Galgal5 compared with Galgal4 is the different read length used when the gene model was established. This change improved inaccurate gene annotations, especially the structure of isoforms, in existing short-read based gene models through an isoform sequencing technique using the Pacific Biosciences (PacBio) long reads. Furthermore, PacBio long-read sequencing technology makes it possible to establish lincRNAs, which is important in developmental biology [14]. Given that our data were not only an early developmental sample of a chicken but also a sample of all types of RNAs, this must be considered when quantifying gene expression levels in the RNA-seq pipeline. Therefore, we decided to quantify the expression level of the entire transcriptome using the two different versions of the genome builds, and then compared the results to examine the differences. In the alignment step, HISAT2 ver. 2.0.0 [15] was used with the “--rna-strandness RF” option to consider a stranded specific library. As a result, an average of 76.07 and 73.27% mapping rates were observed in Galgal4 and Galgal5, respectively, in the 21 bulked embryo samples and 84.41 and 84.28% were observed in the nine single embryo or cell samples (Table 1). For Galgal4 and Galgal5, the average observed difference in the mapping rate between the bulked and single embryo samples was 8.35 and 11%, respectively. We suspected that this difference in mapping rates was caused by the diversity of genetic information. Since transcriptome data generated using single embryo sequencing technology contains only genetic information for a single entity, it is assumed that the mapping rate is increased by alleviating the heterogeneity problem derived from various genetic backgrounds. We also observed small differences in the average mapping rates (0.028 and 0.001% were decreased in Galgal5, for the bulked and single embryo samples, respectively), which implies that there are no large differences between the two genome builds at the DNA level. Following the alignment step using the two different versions of the genome builds,

alignment files (.SAM files) were converted into binary alignment files (.BAM) using SAMtools ver. 1.4.1 [16]. Based on the alignment files, the gene expression levels (number of mapped reads) were quantified using HTSeq-count [17] with the Ensembl gene annotation files corresponding to the genome builds (Ensembl release 85 for Galgal4 and 86 for Galgal5). As a result, the number of mapped reads was quantified in each pipeline and 17,108 and 26,102 genes were annotated in the Galgal4 and Galgal5 genome builds, respectively.

Comparison of the gene expression patterns between Galgal4 and Galgal5 in chicken early embryo samples

Based on the mapped-count matrix of the genome builds and the Ensembl annotation, we systematically investigated how many and which types of genes differed between the two genome builds. First, we found that many genes were differentially annotated in each build in terms of their Ensembl IDs (Fig. 3a). Of the 17,108 and 26,102 annotated genes in Galgal4 and Galgal5, respectively, only 11,451 Ensembl IDs were shared by both annotations, while 5,657 and 14,651 Ensembl IDs were annotated only in the respective builds. Next, we compared the two genome builds based on the genes actually expressed in the early embryo samples of chickens. For this comparison, we filtered out genes with no mapped counts across all 30 RNA-seq samples. As a result, 901 and 3,849 genes were filtered out in the raw gene annotations of Galgal4 and Galgal5, respectively (*i.e.*, 16,207 and 22,253 genes remained). Because the same pattern of results was observed when validated with the filtered Ensembl IDs (Fig. 3b), we then examined which RNAs produced the difference between Galgal4 and Galgal5. As a result, many lincRNAs and protein-coding genes were newly identified in Galgal5 and confirmed to be expressed in early chicken embryos (Fig. 3c and Table 2). With the development of sequencing technology, lincRNA has been added to over 5,166 new genes, and it was confirmed that it is actually expressed in our data. Unlike

lincRNA, which was unilaterally added to Galgal5, there were many changes in protein-coding genes (Table 2). A total of 4,892 protein-coding genes were discarded, while 5,613 were added in the new version of the gene annotation (based on the Ensembl ID matching). Since there is still a lack of empirical evidence and practical discussion of the validity of both gene models, it is impossible to determine which genome build is correct for quantifying gene expression in our study. However, we expect to contribute to further studies by providing the entire transcript expression metrics for early embryos of chickens in both builds. Finally, correlations between the 30 samples were examined based on the quantified expression of 11,001 genes common to the gene annotations of these two builds (Fig. 3d). Based on bulked embryo sequencing, high correlations (≥ 0.9) were observed between Galgal4 and Galgal5, except for the oocyte and zygote. In comparison, single embryo and/or cell sequencing showed the high correlation between Galgal4 and Galgal5 including the oocyte and zygote. This demonstrates the excellent reproducibility of the data produced based on the unique genetic background of a single experimental subject. Most of the embryonic transcriptome data generated to date have involved pooling problems and we expect to be able to perform more sophisticated downstream analysis using single embryo and/or cell sequencing, which is now possible due to technological developments.

Comparison of bulked embryo sequencing and single embryo and/or cell sequencing with chicken early embryos

To investigate the differences between the two technologies more systematically, multidimensional scaling analysis was performed using information from 30 RNA-seq samples in two gene expression matrixes: Galgal4 and Galgal5. All of the samples in both gene expression matrixes clearly clustered according to their developmental stage, except for the zygote, EGK.I, and EGK.III (Fig. 4). This means that although there are morphological differences, there is no transcriptome change during the early embryonic development of the

chicken for a specific time after zygotic gene activation. In fact, the time from the zygote to EGK.III is also very short. While most of the patterns seem to be concordant between Galgal4 and Galgal5, distinct differences were observed between the bulked and single embryo RNA-seq techniques for the oocyte and zygote samples based on the Galgal5 gene expression matrix. However, no difference was detected between the two techniques for the EGK.X samples, which is presumably due to the difference between the bulked and single cells because we performed single embryo RNA-seq for the oocyte, zygote, and EGK.X stages. The RNA samples from the oocyte and zygote were derived from a single cell, whereas those from EGK.X were derived from bulked cells. As we have already examined the difference in gene annotation between Galgal4 and Galgal5, more than 10,000 genes have been changed, which includes both protein-coding genes and lincRNAs. Of these changes, 5,166 newly added lincRNAs may be a major factor causing this difference because lincRNA plays an important role in the zygote as an epigenetic marker in both humans and mice, which have been subjected to lincRNA annotation and early embryonic transcription studies. Furthermore, epigenetic markers are very sensitive, exhibiting subject- or cell-specific characteristics. Therefore, our RNA-Seq data based on the single embryo and cell technique for oocytes and zygotes is more accurate than ordinary RNA-Seq data because it eliminates epigenetic and genetic pooling effects. For example, bulked zygote samples were separated from the cluster of EGK.I and EGK.III samples in a multidimensional scaling (MDS) analysis based on the Galgal5 gene matrix, whereas there was no difference in the Galga4 gene expression matrix (Fig. 4, right panel). This shows that quantifying gene expression using the standard RNA-Seq pooled embryo sequencing technique can lead to false positive results regarding differentially expressed genes.

In summary, we produced the first whole transcriptome sequences of pre-oviposited early chicken embryos based on standard RNA-Seq and single embryo sequencing techniques. We then quantified and compared gene expression using the standard gene

annotation used for the chicken and a new chicken gene annotation based on the advanced long-read sequencing technique. As a result, we not only demonstrated the accuracy of RNA-Seq data based on single embryo or cell sequencing but also successfully quantified 5,166 lincRNAs in the new chicken gene model, for the pre-oviposited early chicken embryo. We expect that the transcriptome sequences of pre-oviposited early chicken embryos will fill the gap in comparative developmental and evolutionary studies of vertebrates as a valuable resources and provide comprehensive knowledge of early avian embryogenesis. Furthermore, the oocyte and early chicken embryos express numerous types of RNA, including mRNA and lincRNA, so our dataset should help to establish novel transcript and gene annotations for the chicken reference genome. Our large dataset should also be useful for future studies of avian and comparative genomics because the data were generated using the latest sequencing platform and whole transcriptome sequencing enabling the characterization of all RNA transcripts, including primary transcripts, regardless of polyadenylation.

Availability of supporting data

The bulked and single embryo RNA-Seq data have been deposited in the NCBI GEO database (GSE86592 and GSE100798, respectively). Supporting data including pre-processed gene expression levels are also available in the GigaScience database (GigaDB).

Abbreviations

EGK: Eyal-giladi and Kochav; lincRNA: long intergenic non-coding RNA; MDS: multidimensional scaling; PacBio: Pacific Biosciences; RNA-Seq: RNA-sequencing; WL: white leghorn.

Experimental animals and animal care

The care and experimental use of chickens were approved by the Institute of Laboratory Animal Resources, Seoul National University (SNU-150827-1). Chickens were maintained according to a standard management program at the University Animal Farm, Seoul National University, Korea. The procedures for animal management, reproduction and embryo manipulation adhered to the standard operating protocols of our laboratory.

Competing interests

The authors declare that they have no competing interests.

Funding

This work was supported by the National Research Foundation of Korea (NRF) grant funded by the Korea government (MSIP) (No. NRF-2015R1A3A2033826).

Authors' contributions

YSH and JYH conceived and designed the experiments. YSH, HJC, and SKK collected embryos. YSH prepared RNA samples and generated whole-transcriptome RNA-seq reads. YSH, MS, HK, and JYH analyzed and wrote the manuscript. All authors read and edited the manuscript.

Author details

[1]Department of Agricultural Biotechnology and Research Institute of Agriculture and Life Sciences, Seoul National University, Seoul 08826, Korea. [2]CHO&KIM genomics, SNU Research Park, Seoul National University Mt.4-2, Seoul 08826, Korea. [3]Channing Division of Network Medicine, Harvard Medical School and Brigham and Women’s Hospital, Boston, Massachusetts, USA. [4]Institute for Biomedical Sciences, Shinshu University, Minamiminowa, Nagano 399-4598, Japan.

References

1. Kispert A, Ortner H, Cooke J and Herrmann BG. The chick Brachyury gene: developmental expression pattern and response to axial induction by localized activin. Dev Biol. 1995;168 2:406-15. doi:10.1006/dbio.1995.1090.
2. Streit A, Berliner AJ, Papanayotou C, Sirulnik A and Stern CD. Initiation of neural induction by FGF signalling before gastrulation. Nature. 2000;406 6791:74-8. doi:10.1038/35017617.
3. Skromne I and Stern CD. Interactions between Wnt and Vg1 signalling pathways initiate primitive streak formation in the chick embryo. Development. 2001;128 15:2915-27.
4. Torlopp A, Khan MA, Oliveira NM, Lekk I, Soto-Jimenez LM, Sosinsky A, et al. The transcription factor Pitx2 positions the embryonic axis and regulates twinning. Elife. 2014;3:e03743. doi:10.7554/eLife.03743.
5. Eyal-Giladi H and Kochav S. From cleavage to primitive streak formation: a complementary normal table and a new look at the first stages of the development of the chick. I. General morphology. Dev Biol. 1976;49 2:321-37.
6. Kochav S, Ginsburg M and Eyal-Giladi H. From cleavage to primitive streak formation: a complementary normal table and a new look at the first stages of the development of the chick. II. Microscopic anatomy and cell population dynamics. Dev Biol. 1980;79 2:296-308.
7. Park HJ, Park TS, Kim TM, Kim JN, Shin SS, Lim JM, et al. Establishment of an in vitro culture system for chicken preblastodermal cells. Mol Reprod Dev. 2006;73 4:452-61. doi:10.1002/mrd.20441.
8. Jarvis ED, Mirarab S, Aberer AJ, Li B, Houde P, Li C, et al. Whole-genome analyses resolve early branches in the tree of life of modern birds. Science. 2014;346 6215:1320-31. doi:10.1126/science.1253451.
9. Zhang G, Li C, Li Q, Li B, Larkin DM, Lee C, et al. Comparative genomics reveals insights into avian genome evolution and adaptation. Science. 2014;346 6215:1311-20. doi:10.1126/science.1251385.
10. Zhang G, Li B, Li C, Gilbert MT, Jarvis ED, Wang J, et al. Comparative genomic data of the Avian Phylogenomics Project. Gigascience. 2014;3 1:26. doi:10.1186/2047-217X-3-26.
11. Lee HC, Choi HJ, Park TS, Lee SI, Kim YM, Rengaraj D, et al. Cleavage events and sperm dynamics in chick intrauterine embryos. PLoS One. 2013;8 11:e80631. doi:10.1371/journal.pone.0080631.
12. Bolger AM, Lohse M and Usadel B. Trimmomatic: a flexible trimmer for Illumina sequence data. Bioinformatics. 2014:btu170.
13. Andrews S. FastQC: A quality control tool for high throughput sequence data. Reference Source. 2010.
14. Durruthy-Durruthy J, Sebastiano V, Wossidlo M, Cepeda D, Cui J, Grow EJ, et al. The primate-specific noncoding RNA HPAT5 regulates pluripotency during human preimplantation development and nuclear reprogramming. Nat Genet. 2016;48 1:44-52. doi:10.1038/ng.3449.
15. Jeong H, Sung S, Kwon T, Seo M, Caetano-Anollés K, Choi SH, et al. HGTree: database of horizontally transferred genes determined by tree reconciliation. Nucleic acids research. 2015;44 D1:D610-D9.
16. Li H, Handsaker B, Wysoker A, Fennell T, Ruan J, Homer N, et al. The sequence alignment/map format and SAMtools. Bioinformatics. 2009;25 16:2078-9.

17. Love MI, Huber W and Anders S. Moderated estimation of fold change and dispersion for RNA-seq data with DESeq2. Genome biology. 2014;15 12:550.

Table 1. Summary statistics of the RNA-seq processing

Bulked embryonic sequencing				
**Samples**	**Surviving reads**	**Surviving rates**	**Mapping rates (Galgal4)**	**Mapping rates (Galgal5)**
Oocyte_S1_Bulked	56024575	94.81%	82.73%	84.32%
Oocyte_S2_Bulked	56043780	94.14%	82.77%	79.54%
Oocyte_S3_Bulked	59498675	95.54%	82.16%	82.39%
Zygote_S1_Bulked	53378148	96.74%	82.43%	85.89%
Zygote_S2_Bulked	53999584	96.77%	82.19%	79.86%
Zygote_S3_Bulked	50027929	98.02%	80.90%	87.58%
EGK.I_S1_Bulked	56909314	97.36%	74.55%	70.70%
EGK.I_S2_Bulked	61447014	97.94%	73.24%	68.64%
EGK.I_S3_Bulked	50188847	96.80%	81.34%	77.01%
EGK.III_S1_Bulked	60876681	97.30%	76.06%	69.37%
EGK.III_S2_Bulked	56357690	97.90%	75.20%	70.47%
EGK.III_S3_Bulked	45715485	98.02%	75.30%	70.38%
EGK.VI_S1_Bulked	62075038	97.53%	71.14%	63.68%
EGK.VI_S2_Bulked	65223164	97.77%	80.95%	72.89%
EGK.VI_S3_Bulked	49604292	98.16%	75.12%	69.22%
EGK.VIII_S1_Bulked	67401388	97.35%	70.10%	67.32%
EGK.VIII_S2_Bulked	56396268	96.82%	66.53%	60.37%
EGK.VIII_S3_Bulked	71309063	97.44%	70.68%	70.70%
EGK.X_S1_Bulked	67730502	95.70%	72.24%	69.29%
EGK.X_S2_Bulked	74109500	95.02%	70.64%	69.62%

EGK.X_S3_Bulked	63225919	94.65%	71.13%	69.51%
**Average**	58930612.19	0.967514286	0.760666667	0.732738095
**Single embyonic or cell sequencing**				
Oocyte_S1_SingleCell	23558381	86.61%	86.28%	86.67%
Oocyte_S2_SingleCell	53963445	84.75%	85.95%	85.86%
Oocyte_S3_SingleCell	24660386	84.95%	84.95%	84.33%
Zygote_S1_SingleEmbryo	31742857	87.17%	84.32%	84.40%
Zygote_S2_SingleEmbryo	91033778	85.72%	76.59%	76.15%
Zygote_S3_SingleEmbryo	27687195	87.60%	86.02%	85.96%
EGK.X_S1_SingleEmbryo	30914824	86.41%	83.67%	83.16%
EGK.X_S2_SingleEmbryo	47159061	86.29%	88.38%	89.10%
EGK.X_S3_SingleEmbryo	29006546	85.94%	83.57%	82.86%
**Average**	39969608.11	0.8616	0.844144444	0.842766667

Table 2. Comparison of Galgal4 and Galgal5 gene annotations

RNAs	Annotated in Galgal4 only	Commonly annotated	Annotated in Galgal5 only
lincRNA	0	0	5,166
miRNA	204	487	253
misc_RNA	15	71	43
Mt_rRNA	2	0	2
Mt_tRNA	10	0	14
protein_coding	4,892	10,213	5,613
pseudogene	29	10	25
rRNA	6	8	58
scaRNA	0	0	4
snoRNA	41	172	44
snRNA	7	40	30
Total	5,206	11,001	11,252

Figure legends

Fig. 1. The bulked and single embryonic RNA-Sequencing (RNA-Seq) in early chicken development. a) The diagram of bulked embryonic RNA-seq. Total 137 pre-oviposited embryos were collected. Each replicate contains from three to ten embryos pooled. The bulked embryo RNA-Seq was performed in triplicate. b) The diagram of single embryonic RNA-Seq. The single oocyte, zygote, and Eyal-giladi and Kochav stage X (EGK.X) blastoderm were obtained from one hen simultaneously. Samples was collected from three hen. Single embryo was sequenced as one replicate and each stage consists of triplicated embryos from three hen, respectively. c) The representative stages of chicken early embryos used for RNA-Seq. Dorsal views of whole embryos from the oocyte to EGK.X are shown. A germinal vesicle oocyte in the ovary and fertilized zygote in the magnum without cleavage were obtained. The intrauterine embryos were obtained 5.5 (EGK.I), 8.5 (EGK.III), 15.5 (EGK.VI), and 20.5 (EGK.VIII) h after fertilization. The EGK.X embryo was obtained after oviposition. Scale bar, 1000 µm.

Fig. 2. Collection of bulked and single embryos during early chicken development. a) The number of embryos in each sample. b) The RNA concentration and c) total amount of RNA for each stage used in RNA-Seq. d) The estimated total RNA per embryo in the bulked samples and the total amount of RNA in a single embryo. The RNA concentration, amount of RNA, and total RNA per embryo did not differ significantly among the groups (Kruskal–Wallis test, $P > 0.05$).

Fig. 3. Comparison of two different builds of gene annotation for the early chicken embryo samples. a) Using the Ensembl annotation with the two different genome builds, annotated genes were compared based on the Ensembl ID. As a result, 5,657 and 14,651 Ensembl IDs were identified in Galgal4 and Galgal5, respectively, while 11,451 Ensembl IDs are common

to the two different annotations. b) Based on the expressed genes at any stage of the chicken early embryos, the gene lists were compared between Galgal4 and Galgal5. c) Investigation of the change in annotated genes in Galgal5 among genes expressed in early chicken embryos. As a result, a large number of lincRNAs was added as new features in Galgal5. d) A correlation analysis of the total gene expression based on 11,001 common annotated genes shared between Galgal4 and Galgal5.

Fig. 4. Multidimensional scaling plots based on all annotated genes in Galgal4 and Galgal5. The gene expression patterns of early chicken embryos quantified based on Galgal4 were clearly differentiated by developmental stage regardless of the sequencing technique used. In comparison, there was a difference between the bulked and single embryo sequencing techniques in the oocyte and zygote in Galgal5.

Figure 1

Click here to download Figure Fig1.pdf

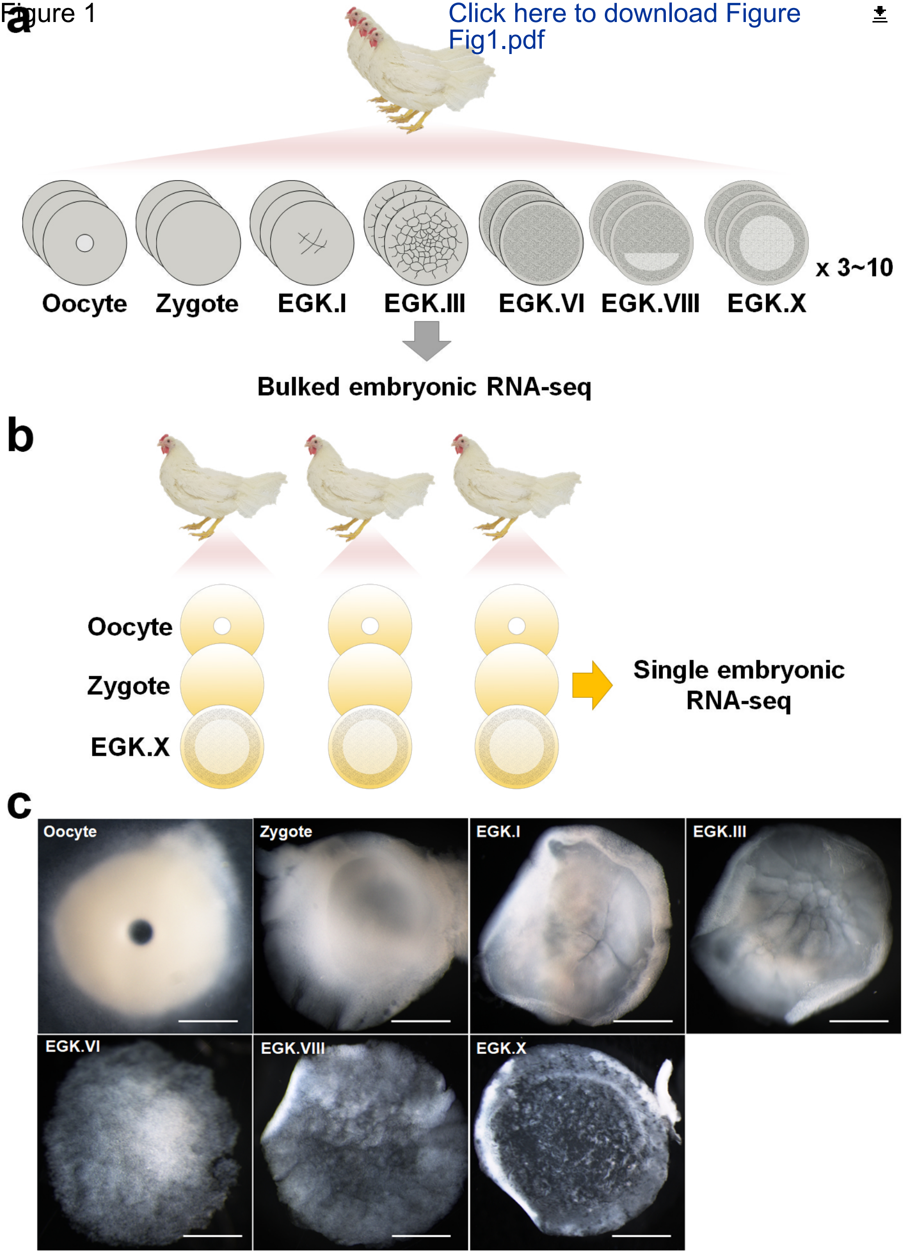

Figure 2

Click here to download Figure Fig2.pdf

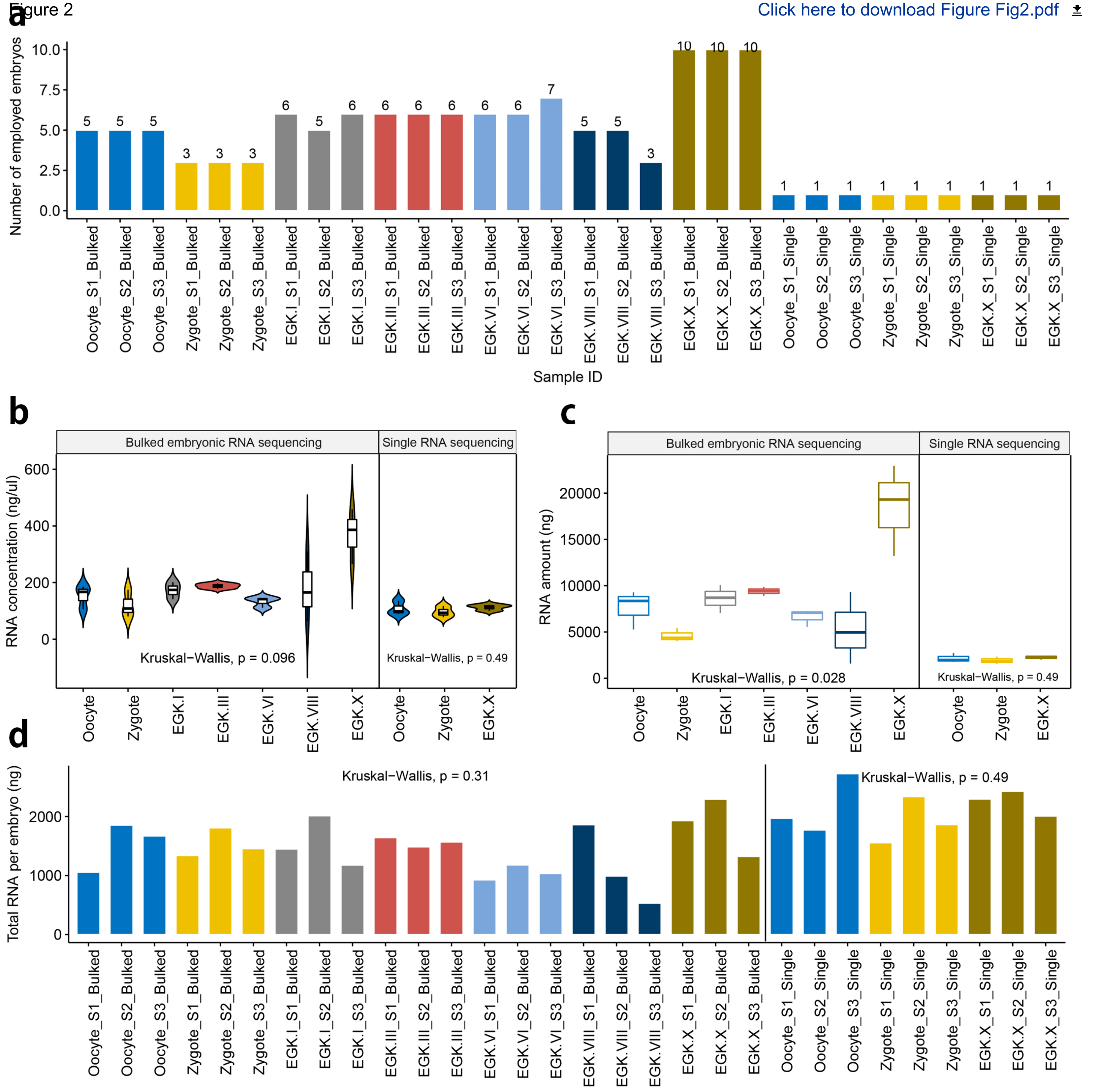

Figure 3

Click here to download Figure Fig3.pdf

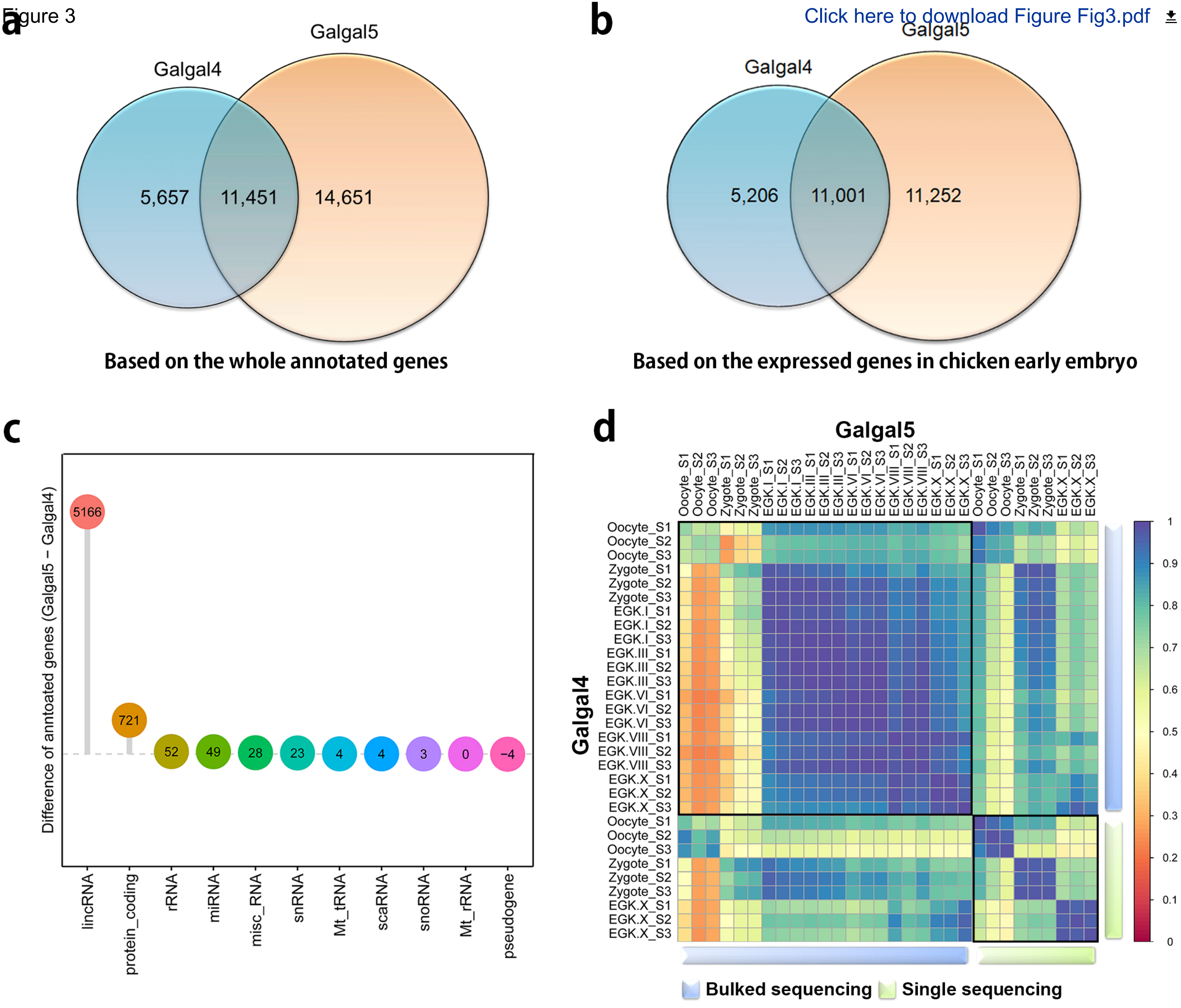

Figure 4

Click here to download Figure Fig4.pdf

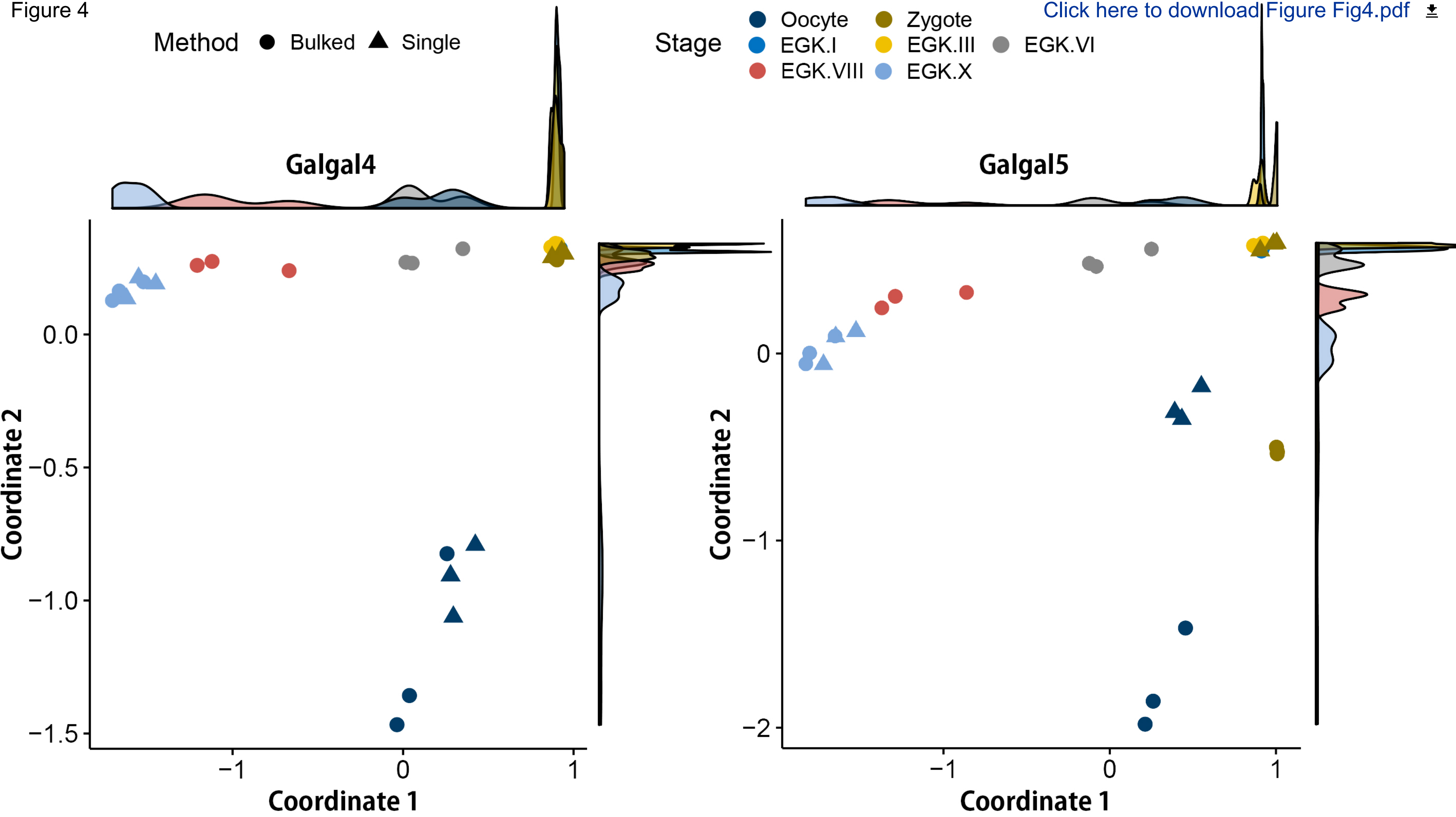

Supplement: GIGA-D-17-00277_Original_Submission.pdf [file giy030_giga-d-17-00277_original_submission.pdf]
