# Supplementary material for: The first whole transcriptomic exploration of pre-oviposited early chicken embryos using single and bulked embryonic RNA-sequencing
Source: Gigascience. 2018 Mar 24;7(4):giy030. doi: 10.1093/gigascience/giy030 (PMC5893961; doi:10.1093/gigascience/giy030)
Supplement: Reviewer_2_Original_Submission_Attachment_Enke_review-GIGA-D-17-00277.pdf [file giy030_reviewer_2_original_submission_attachment_enke_review-giga-d-17-00277.pdf]

Enke Peer review: GigaScience - GIGA-D-17-00277

Hwang and coauthors report their RNA sequencing data of pre-oviposited early chicken embryos. The authors used single cell as well as standard whole tissue RNA-seq analysis and also assess differences between gene annotation in the two most recent chicken genome builds. Given the wide usage of the chicken embryo as a model system to study vertebrate development, this contribution is very important to the research community. I have several issues for the authors to consider revising.

Major Concerns:

1. Quality of total RNA was assessed using several methods including an Agilent Bioanalyzer (lines 97-100). The RNA integrity number (RIN) should be reported for all samples. Typically, RNA samples with a RIN ≤ 7 are not suitable for accurate RNA-seq analysis.

2. The methodology for Illumina sequencing library preparation is insufficiently reported (lines 125-129). More detail should be added here including the method of transcript enrichment and average size of library fragments.

3. Table 1 demonstrates the number of reads that passed Trimmomatic filtering, however, representative FastQC plots such as per bas and/or per sequence quality plots should be shown in the main text or supplement to demonstrating the quality of the data.

4. All settings for Trimmomatic filtering software (line 135) HISAT2 alignment software (line 155), and HTSeq-count transcript quantification software (line 171) should be reported.

5. Figure 3 alludes to interesting differences in gene annotation between Galgal4 and Galgal5 genome builds but does not report what these newly annotated transcripts are in the updated annotation. A table of genes/transcripts represented in Fig3a-c should be included.

6. Figure 4 alludes to interesting differences in gene expression between developmental stages but there are no reports on what these genes are in the main body of the paper. An additional figure or table should be added to report several aspects of differential gene expression between embryo stages.

Review questions:

Overall recommendation: minor revision
Level of interest: An article whose findings are important to those with closely related research interests
Quality of written English: Acceptable
Declaration of competing interests: 'I declare that I have no competing interests
I agree to the open peer review policy of the journal

*Please note that I do not want my contact info to be added to Publons.
