# Supplementary material for: The first whole transcriptomic exploration of pre-oviposited early chicken embryos using single and bulked embryonic RNA-sequencing
Source: Gigascience. 2018 Mar 24;7(4):giy030. doi: 10.1093/gigascience/giy030 (PMC5893961; doi:10.1093/gigascience/giy030)
Supplement: Table S1 [file giy030_supp.docx]

Table S1. rRNA ratio during pre-ovipositional development and RNA integrity number (RIN) of the RNA-seq samples

| Bulked embryonic sequencing | | | | | |
| --- | --- | --- | --- | --- | --- |
| **Samples** | **rRNA ratio (28s : 18s)** | **Mean of rRNA ratio** | | **Standard deviation of rRNA ratio** | **RIN** |
| Oocyte_S1_Bulked | 1.832 | 1.895 | | 0.0807 | 8.5 |
| Oocyte_S2_Bulked | 1.986 |  |  |  | 9.3 |
| Oocyte_S3_Bulked | 1.867 |  |  |  | 9.2 |
| Zygote_S1_Bulked | 1.558 | 1.527 | | 0.0310 | 7.9 |
| Zygote_S2_Bulked | 1.496 |  |  |  | 6.5 |
| Zygote_S3_Bulked | 1.526 |  |  |  | 6.6 |
| EGK.I_S1_Bulked | 1.458 | 1.527 | | 0.0627 | 6.7 |
| EGK.I_S2_Bulked | 1.58 |  |  |  | 7.2 |
| EGK.I_S3_Bulked | 1.544 |  |  |  | 7.5 |
| EGK.III_S1_Bulked | 1.573 | 1.594 | | 0.0947 | 7.6 |
| EGK.III_S2_Bulked | 1.697 |  |  |  | 6.5 |
| EGK.III_S3_Bulked | 1.511 |  |  |  | 7.4 |
| EGK.VI_S1_Bulked | 1.54 | 1.506 | | 0.0368 | 7.4 |
| EGK.VI_S2_Bulked | 1.467 |  |  |  | 6.6 |
| EGK.VI_S3_Bulked | 1.511 |  |  |  | 6.5 |
| EGK.VIII_S1_Bulked | 1.783 | 1.807 | | 0.0463 | 7.6 |
| EGK.VIII_S2_Bulked | 1.777 |  |  |  | 7.2 |
| EGK.VIII_S3_Bulked | 1.86 |  |  |  | 6.8 |
| EGK.X_S1_Bulked | 1.828 | 1.939 | | 0.0993 | 7.9 |
| EGK.X_S2_Bulked | 2.02 |  |  |  | 7.9 |
| EGK.X_S3_Bulked | 1.968 |  |  |  | 7.9 |
| **Single embryonic or cell sequencing** | | | | | |
| **Samples** | | | **RIN** | | |
| Oocyte_S1_SingleCell | | | 8.6 | | |
| Oocyte_S2_SingleCell | | | 8.5 | | |
| Oocyte_S3_SingleCell | | | 8.4 | | |
| Zygote_S1_SingleEmbryo | | | 6.5 | | |
| Zygote_S2_SingleEmbryo | | | 6.6 | | |
| Zygote_S3_SingleEmbryo | | | 6.3 | | |
| EGK.X_S1_SingleEmbryo | | | 7.8 | | |
| EGK.X_S2_SingleEmbryo | | | 7.2 | | |
| EGK.X_S3_SingleEmbryo | | | 7.8 | | |
