# Supplementary material for: The first whole transcriptomic exploration of pre-oviposited early chicken embryos using single and bulked embryonic RNA-sequencing
Source: Gigascience. 2018 Mar 24;7(4):giy030. doi: 10.1093/gigascience/giy030 (PMC5893961; doi:10.1093/gigascience/giy030)
Supplement: Response_to_Reviewer_Comments_Original_Submission.pdf [file giy030_response_to_reviewer_comments_original_submission.pdf]

Author's Response To Reviewer Comments

Dear Editor,

Herewith we are submitting our revised manuscript entitled “The first whole transcriptomic exploration of pre-oviposited early chicken embryos using single and bulked embryonic RNA-sequencing” (Manuscript ID: GIGA-D-17-00277).

We would like to express our sincere gratitude to the editor and reviewers who handled this manuscript. We also appreciate the positive and thoughtful comments of the both reviewers who have acknowledged the value of the data submitted. We believe that their comments have greatly improved the reproducibility and readability of our work, especially about the quality control and methodologies of our data. This manuscript was substantially revised according to all of the reviewers’ comments. We also prepared a point-by-point response to the reviewer’s comments and submitting herewith.

We are looking forward to meet editorial and reviewer suggestion regarding this revised manuscript.

Sincerely yours,

Jae Yong Han, Ph.D.

The authors are very grateful for the response on the originally submitted manuscript. In accordance to the comments by the reviewers, we have provided point-to-point responses to all of your comments. We believe that this revision and our responses will satisfy your point of view on our manuscript.

The authors present a new dataset they generated with first analytical steps. Emphasizing that chicken embryos are a very useful model to study because, in part of the accessibility of the embryo in the egg and thus to key steps of the development. However studies so far are focusing their efforts on the accessible stages of the development, i.e. those occurring in the egg and we are missing information on the development stages happening before oviposition.
The authors produced RNA seq data of those pre-oviposition stages thus giving access to the first expression map of chicken embryos at those early stages in development. Aware of technical biases, the authors sequenced the data from 2 different sources, single embryo vs bulked embryos. This gives an interesting overview of potential differences and strength of both approaches in the context of sequencing those very early stages. Also aware that researchers in the chicken community are still using Galgal4 reference build for comparison purposes, the authors aligned their sequenced data on the 2 most recent builds Galgal4 and Galgal5. This allows to appreciate intrinsic differences between both builds.

This dataset is of interest for any researcher in embryology, in chicken or not, that would like to access expression data of early stages of development.
The authors made a good job at controlling the quality their data for the most part. I would

definitely recommend to publish this work provided some minor additions/modifications.

1. L55: precise this first set is bulked
Response: We are thankful of the comments for improving our manuscript by the reviewer. As the reviewer's suggestion, we added "bulked" in that sentence (Line 54).

2. L79: stages could be put in chronological order
Response: As the reviewer pointed out, we re-ordered to "the oocyte, the zygote, EGK.I, EGK.III, EGK.VI, EGK.VIII and EGK.X" (Line 78-79).

3. L81: to what extend this technique could have an impact on the embryo integrity, and consequently RNA quality? More generally, for the bulked sequences, how many hens were used? What genetic background are they? Could the authors justify the choice of choosing different animals for the bulk sequencing and the same ones for different stages for the single embryo sequencing?
Response: The embryo integrity and RNA quality are not affected by the abdominal massage technique for harvesting early embryos in chicken. Using these embryos, we can perform the downstream experiments such as in situ hybridization, qRT-PCR, and RNA sequencing. Also, we used total 137 hens for bulked embryonic sequencing after egg-laying time checked.
On the other hands, White leghorn flock we used is registered in Domestic Animal Diversity Information System (DAD-IS; http://www.fao.org/dad-is/) as "White Leghorn SNU" and they have been systematized since they were brought from National Institute of Animal Science in 1992. Thus, the chickens for bulked embryo and single embryo are considered to be the closed population with a genetically similar background.
Nevertheless, the expression profiles were shown to be different between bulked and single oocyte and zygote based on Galgal5. This seems to be caused by individual variation and maternal effects in terms of gene expressions during very early stages such as oocyte and zygote, but not in EGK.X. In addition, we changed the words, "genetic information" and "genetic backgrounds" into "the individual gene expression diversity" (Line 170, 172), "its own gene expression" (Line 174), and "various individuals" (Line 176) for not making the readers confused the variation of gene expression with the variation of genetic background.

4. L90: what is the rationale behind the number of embryos pooled together at each stage? Why did the authors chose a higher number for the latest stage?
Response: We thank the reviewer for pointing out this important issue. When we did sampling oocyte, zygote, and intrauterine stages, firstly we checked the stage of embryo morphologically. Immediately after identifying the stage of the embryos, we pooled at least three embryos per one replicate in each stage. In this procedure, it is difficult to use the exact number of samples in all stages, owing to the limited acquisition of intrauterine embryos. In the case of EGK.X which is at oviposition, we could easily obtain a relatively larger number of embryos as one replication.

5. L123: It would be extremely interesting to have on idea of the RNA quality. What is the RIN for each sequenced sample? This information might be useful to interpret some of the further results.
Response: As the reviewer's question, we prepared rRNA ratio during pre-ovipositional development and RIN of all samples in revised Table S1: Additional file 1. RIN number below 7

were observed from zygote to EGK.VIII stage in few samples although same RNA isolation procedure was applied in oocyte and EGK.X. This is because of common phenomenon that rRNA ratio (28s: 18s) is lower than 1.8 from zygote to EGK.VIII stages, not caused by RNA quality. The low levels of 28s rRNA prior to maternal-to-zygotic transition (MZT) were generally found during early embryonic development. In chicken, the relative amount of 28s rRNA was reduced markedly after the zygote and recovered gradually after EGK.VIII at MZT occurring (Hwang et al., FASEB J 2017), like as bovine until morula stage (Gilbert et al., Mol Reprod Dev 2009). We also added the related sentences to the revised manuscript (Line 100-103 and Line 123-124).

6. L129: replace with 150bp paired-end reads
Response: According to reviewer's suggestion, we replaced it (Line 134-135).

7. L135: What are the criteria used for filtering after FastQC? We would need some more information to help explain the differences in quality between single embryo and bulked.
Response: Thanks for good suggestion. Here, after Trimmomatic, minimum read length > 75 bp and Phred score > 30 were checked using FastQC (Line 142-144). In addition, we have uploaded all FastQC results in GigaDB including figures and data derived from FastQC.

Location of directory in the GigaDB:
(1) /0.FastQC/1.Bulked_embryo_fastqc.zip: FastQC results for fastq files from bulked RNA-seq data
(2) /0.FastQC/2.Single_Embryo_fastqc.zip: FastQC results for fastq files from single embyonic RNA-seq data

8. L161: This statement depends on what is the genetic diversity of hens used in the study and how close the hens are from the reference genome. The authors could give other clues to explain those differences. What is the duplication rate? What is the proportion of reads that are uniquely mapped?
Response: Thank you very much for providing us with a clue to our hypothesis. In revised Table 1, deduplicated percentage derived from FastQC and uniquely mapped reads derived from HISAT2 log were added based on the reviewer's comment. First, we observed significant differences between bulk and single RNA-seq data in terms of duplication rate. In the bulked samples, 31.3% and 42.96% were observed for read 1 and read 2, respectively (In case of, single embryonic sample, 44.78% and 49.33% were observed for read 1 and read 2, respectively). In other words, this result showed a higher read duplication rate in RNA-seq performed with a single embryo sample, demonstrating that there is lower expressional diversity in single embryonic samples. This result has been added to the result body in revised manuscript (Line 170-173).

9. L166: rephrase this sentence
Response: We have rephrased it and corrected the error (0.028 and 0.001 to 2.79 and 0.14) in this sentence (Line 176-180).

10. L190: replace "which RNAs" with "which RNA category" or "which RNA type"
Response: According to reviewer's suggestion, we replaced it (Line 203).

11. L194: what is the threshold of expression used to call a gene expressed?
Response: Here, we just used mapped reads on the reference genome across all samples. Based on the read count of each genes, we designated them as expressed genes (Line 208).

12. L205: this might be linked to the quality of those samples. Is it harder to extract quality RNA from very early stages?
Response: As shown in revised Table S1: Additional file 1, the extracted RNA from these embryos have good quality. Thus, RNA quality does not seem to be related to the different correlation between Galgal4 and Galgal5.

13. L242: The author might want to moderate this sentence. Here bulked embryos were from different genetic background and extracted with a very specific technic. We would like to know to what extend those choices can impact the quality of the data as well. It might not be solely due to the pooling process.
Response: As the above answers, the sample quality does not seem to be relevant to these effects. In terms of the evidences we have found, the pooling effect from the individual gene expression diversity and the difference of gene annotations could impact on such results between oocyte and zygote (Line 257-258).

14. Table1: "surviving": would suggest to rephrase
Response: We have corrected “Surviving reads” to “QC passed reads“ and “Surviving rates” to “QC passing rate” in revised Table 1.

15. Figure 2b is it a pairwise comparison? What difference are you testing here?
Response: Here, the null hypothesis assumes that the RNA concentration values of all developmental stages are the same, and the alternative hypothesis hypothesized that RNA concentration values differ in at least one stage. In other words, F-test was performed on a statistical model of one-way Analysis of variance (ANOVA) format. Consequently, The RNA concentration, amount of RNA, and total RNA per embryo did not differ significantly among the groups (Line 398-400).

16. Figure3a-b: what is the correspondence between those lists and the annotation? What is the percentage of overlap? In other words, how many of those genes that are expressed differentially between galgal4 et galgal5 are actually the ones that are not annotated in one of the builds?
Response: Thank you for suggesting the issues from different references. It is quite difficult to the answer to the reviewer’s comment. First, we can’t perform analysis identifying differentially expressed genes (DE analysis) between Galgal4 and Galgal5 with the genes in a particular build. Please understand that in order to perform DE analysis between both builds, we can only perform on approximately 11,000 genes that were commonly annotated in both builds. Second, if we perform DE analysis on genes that were commonly annotated in both builds, there is a statistical issue. Currently, RNA-seq statistical analysis is performed on a generalized linear model (GLM). Hypothesis testing for differences between two builds violates the independence assumption in this model. To resolve this issue, generalized linear mixed-effect model (GLMM) should be employed, but this model has not yet been proven as standard method. This question is very interesting, but adding it to a discussion in the main text does not seem to fit the article type of

the Data Note, so it only answers the letter.

17. Figure 3c is redundant with Table 2. Y axis: anntoated —> annotated
Response: First, typo that the reviewer pointed out has been fixed in Fig. 3c. But, Figure 3c and Table 2 are different. In case of revised Table 2, the contents include only annotation comparison between Galgal4 and Galgal5. On the other hand, Figure 3c demonstrates that how many newly annotated genes were actually expressed in terms of mapped reads across all samples. In order to improve the readability for the potential readers, Table 2 and Fig. 3c citation of the main text were modified in Line 204-206.

18. Figure 4: would be interesting to correlate those coordinates with known covariates to show what are in the main axis of variation.
Response: Thanks for reviewer pointing out an important issue. As part of the reviewer, we proceeded with factor analysis through various environmental variables (Batch effects and etc.) with vectors obtained through dimensional reduction. As a result, the variation of the projected vector values of dimension 1 and dimension 2, as mentioned in the text, describes the sub-cluster structure of developmental stages best. First dimension showed that developmental stages progressed in a negative direction during intrauterine development. In case of second dimension, variation of values seem to explain the difference between oocyte and fertilized embryos from zygote to EGK.X (Line 417-420).

Hwang and coauthors report their RNA sequencing data of pre-oviposited early chicken embryos. The authors used single cell as well as standard whole tissue RNA-seq analysis and also assess differences between gene annotation in the two most recent chicken genome builds. Given the wide usage of the chicken embryo as a model system to study vertebrate development, this contribution is very important to the research community. I have several issues for the authors to consider revising.

1. Quality of total RNA was assessed using several methods including an Agilent Bioanalyzer (lines 97- 100). The RNA integrity number (RIN) should be reported for all samples. Typically, RNA samples with a RIN $\leq$ 7 are not suitable for accurate RNA-seq analysis.
Response: We are thankful to the considerate comments by the reviewer. As the reviewer's suggestion, we prepared rRNA ratio during pre-ovipositional development and RIN of all samples in revised Table S1: Additional file 1. RIN number below 7 were observed from zygote to EGK.VIII stage in few samples although same RNA isolation procedure in oocyte and EGK.X. This is because of common phenomenon that rRNA ratio (28s: 18s) is lower than 1.8 from zygote to EGK.VIII stages, not caused by RNA quality. The low levels of 28s rRNA prior to maternal-to-zygotic transition (MZT) were generally found during early embryonic development. In chicken, the relative amount of 28s rRNA was reduced markedly after the zygote and recovered gradually after EGK.VIII at MZT occurring (Hwang et al., FASEB J 2017), like as bovine until morula stage (Gilbert et al., Mol Reprod Dev 2009). We also added the related sentences (Line 100-103 and Line 123-124).

2. The methodology for Illumina sequencing library preparation is insufficiently reported (lines 125- 129). More detail should be added here including the method of transcript enrichment and

average size of library fragments.
Response: As the reviewer's suggestion, we added detailed methods including specific library prep kit product used in this study and the average size of cDNA libraries (Line 131-133)

3. Table 1 demonstrates the number of reads that passed Trimmomatic filtering, however, representative FastQC plots such as per bas and/or per sequence quality plots should be shown in the main text or supplement to demonstrating the quality of the data.
Response: Thanks for good suggestion. Based on suggestion from reviewers, we have uploaded all FastQC results in GigaDB including figures and data derived from FastQC.

Location of directory in the GigaDB:
(1) /0.FastQC/1.Bulked_embryo_fastqc.zip: FastQC results for fastq files from bulked RNA-seq data
(2) /0.FastQC/2.Single_Embryo_fastqc.zip: FastQC results for fastq files from single embyonic RNA-seq data

4. All settings for Trimmomatic filtering software (line 135) HISAT2 alignment software (line 155), and HTSeq-count transcript quantification software (line 171) should be reported.
Response: Thanks for reviewer's suggestion to improve the reproducibility of our paper. Based on the reviewer's comment, we add specific option of used tools as follows:

(1) Trimmomatic ver. 0.33 with "-phred33 and ILLUMINACLIP:/home/Program/Trimmomatic-0.32/adapters/TruSeq3-PE-2.fa:2:30:10 MINLEN:75 option" (Line 141-142).

(2) HISAT2 ver. 2.0.0 [15] was used with the "--rna-strandness RF –x [File name of Galgal4 or Galgal5 reference] -1 [File name of left lead] -2 [File name of right read] 2> [Sample name].log" (Line 163-165).

(3) HTSeq-count [17] with following option, "python -m HTSeq.scripts.count -f bam --stranded=reverse [File name of bam file] [File name of annotation (.GTF file)] > [Output file name] (Line 183-185).

5. Figure 3 alludes to interesting differences in gene annotation between Galgal4 and Galgal5 genome builds but does not report what these newly annotated transcripts are in the updated annotation. A table of genes/transcripts represented in Fig3a-c should be included.
Response: In this study, we just used Galgal4 and Galgal5 reference genome and gene annotations rather than do-novo based assembly. Thus, those annotations have been already provided in Ensembl DB;
(Galgal4, jul2016.archive.ensembl.org/Gallus_gallus/Info/Annotation and
Galgal5, www.ensembl.org/Gallus_gallus/Info/Annotation).
The major difference between two gene builds is shown to be the presence of long non-coding genes and the increased number of gene transcripts.

6. Figure 4 alludes to interesting differences in gene expression between developmental stages but there are no reports on what these genes are in the main body of the paper. An additional figure or table should be added to report several aspects of differential gene expression between

embryo stages.
Response: Since this article type is a Data Note and according to the Criteria of Data Note in GigaScience, we have focused on the results obtained from the pre-processing step as much as possible. As shown in Figure 4, the expression pattern of these genes will vary according to the developmental stages, we expect potential users to unveil their-own downstream analyses based on the raw-count and TMM normalized matrix we provided. Moreover, because of the possibility of duplication of processed data regarding differential gene expression in our further research article, please excuse that we could not provide it completely.
