# Supplementary material for: The first whole transcriptomic exploration of pre-oviposited early chicken embryos using single and bulked embryonic RNA-sequencing
Source: Gigascience. 2018 Mar 24;7(4):giy030. doi: 10.1093/gigascience/giy030 (PMC5893961; doi:10.1093/gigascience/giy030)
Supplement: Reviewer_1_Report_(Original_Submission) -- Laure Fresard — 10 Dec 2017 Reviewed [file giy030_reviewer_1_report_(original_submission).pdf]

Reviewer Report

Title: The first whole transcriptomic exploration of pre-oviposited early chicken embryos using single and bulked embryonic RNA-sequencing

Version: Original Submission **Date:** 10 Dec 2017

Reviewer name: Laure Fresard

Reviewer Comments to Author:

The authors present a new dataset they generated with first analytical steps. Emphasizing that chicken embryos are a very useful model to study because, in part of the accessibility of the embryo in the egg and thus to key steps of the development.
However studies so far are focusing their efforts on the accessible stages of the development, i.e. those occurring in the egg and we are missing information on the development stages happening before oviposition.
The authors produced RNA seq data of those pre-oviposition stages thus giving access to the first expression map of chicken embryos at those early stages in development.
Aware of technical biases, the authors sequenced the data from 2 different sources, single embryo vs bulked embryos. This gives an interesting overview of potential differences and strength of both approaches in the context of sequencing those very early stages.
Also aware that researchers in the chicken community are still using Galgal4 reference build for comparison purposes, the authors aligned their sequenced data on the 2 most recent builds Galgal4 and Galgal5. This allows to appreciate intrinsic differences between both builds.

This dataset is of interest for any researcher in embryology, in chicken or not, that would like to access expression data of early stages of development.
The authors made a good job at controlling the quality their data for the most part. I would definitely recommend to publish this work provided some minor additions/modifications.

Minor points:
L55: precise this first set is bulked
L79: stages could be put in chronological order
L81: to what extend this technique could have an impact on the embryo integrity, and consequently RNA quality?
More generally, for the bulked sequences, how many hens were used? What genetic background are they? Could the authors justify the choice of choosing different animals for the bulk sequencing and the same ones for different stages for the single embryo sequencing?
L90: what is the rationale behind the number of embryos pooled together at each stage? Why did the authors chose a higher number for the latest stage?

L123: It would be extremely interesting to have on idea of the RNA quality. What is the RIN for each sequenced sample? This information might be useful to interpret some of the further results.
L129: replace with 150bp paired-end reads
L135: What are the criteria used for filtering after FastQC? We would need some more information to help explain the differences in quality between single embryo and bulked.
L161: This statement depends on what is the genetic diversity of hens used in the study and how close the hens are from the reference genome. The authors could give other clues to explain those differences. What is the duplication rate? What is the proportion of reads that are uniquely mapped?
L166: rephrase this sentence
L190: replace "which RNAs" with "which RNA category" or "which RNA type"
L194: what is the threshold of expression used to call a gene expressed?
L205: this might be linked to the quality of those samples. Is it harder to extract quality RNA from very early stages?
L242: The author might want to moderate this sentence. Here bulked embryos were from different genetic background and extracted with a very specific technic. We would like to know to what extend those choices can impact the quality of the data as well. It might not be solely due to the pooling process.

Figures/Tables
Table1: "surviving": would suggest to rephrase
Figure 2a: Would be nice to show the impact of number of pooled embryo on the RNA diversity. This number might also explain the bad replication rate for zygote.
Figure 2b is it a pairwise comparison? What difference are you testing here?
Figure3a-b: what is the correspondance between those lists and the annotation? What is the percentage of overlap? In other words, how many of those genes that are expressed differentially between galgal4 et galgal5 are actually the ones that are not annotated in one of the builds?
Figure 3c is redundant with Table 2. Y axis: anntoated —> annotated
Figure 4: would be interesting to correlate those coordinates with known covariates to show what are in the main axis of variation.

As a summary, this is an interesting and useful new dataset. The paper would benefit form adding more details on other potential source of bias to explain the results observed.
In particular:
- justification of the different number of embryos at different stages
- give more details on RNA quality
- give more information on criteria used for expression analysis

The data as been made accessible by the authors.

Level of Interest

Please indicate how interesting you found the manuscript: An article of importance in its field

Quality of Written English

Please indicate the quality of language in the manuscript: Acceptable

Declaration of Competing Interests

Please complete a declaration of competing interests, considering the following questions:

- Have you in the past five years received reimbursements, fees, funding, or salary from an organisation that may in any way gain or lose financially from the publication of this manuscript, either now or in the future?
- Do you hold any stocks or shares in an organisation that may in any way gain or lose financially from the publication of this manuscript, either now or in the future?
- Do you hold or are you currently applying for any patents relating to the content of the manuscript?
- Have you received reimbursements, fees, funding, or salary from an organization that holds or has applied for patents relating to the content of the manuscript?
- Do you have any other financial competing interests?
- Do you have any non-financial competing interests in relation to this paper?

If you can answer no to all of the above, write 'I declare that I have no competing interests' below. If your reply is yes to any, please give details below.

I declare that I have no competing interests

I agree to the open peer review policy of the journal. I understand that my name will be included on my report to the authors and, if the manuscript is accepted for publication, my named report including any attachments I upload will be posted on the website along with the authors' responses. I agree for my report to be made available under an Open Access Creative Commons CC-BY license (http://creativecommons.org/licenses/by/4.0/). I understand that any comments which I do not wish to be included in my named report can be included as confidential comments to the editors, which will not be published.

I agree to the open peer review policy of the journal

To further support our reviewers, we have joined with Publons, where you can gain additional credit to further highlight your hard work (see: https://publons.com/journal/530/gigascience). On publication of this paper, your review will be automatically added to Publons, you can then choose whether or not to claim your Publons credit. I understand this statement.

Yes
